# Cultivating a Meaningful Application of IMFs through Backward Laboratory Course Design

**DOI:** 10.1021/acs.jchemed.3c00810

**Published:** 2024-05-08

**Authors:** Brenda B. Harmon, Deepika Das, Annette W. Neuman, Simbarashe Nkomo, Nichole L. Powell, Austin Scharf

**Affiliations:** † Department of Chemistry, Oxford College of Emory University, Oxford, Georgia 30054, United States

**Keywords:** First-Year Undergraduate, Curriculum, Lab Practical, Practical Exam, Scaffolding Process, Backward Design, Inquiry-Based/Discovery Learning, Student-Centered Learning, Meaningful Learning, Applications of Chemistry

## Abstract

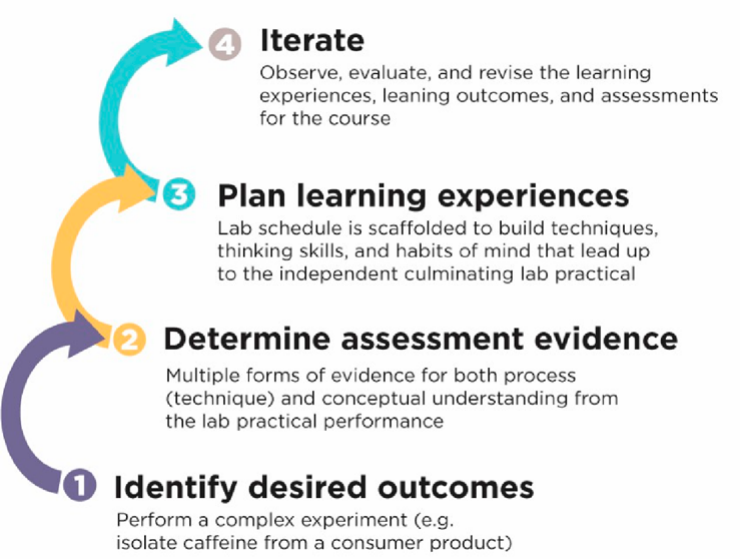

This paper describes the development of a first- and second-year inquiry-based laboratory course focused on the development of a meaningful application of intermolecular forces (IMFs). Instead of broad expository coverage of topics, we used backward design: the techniques and concepts for the course were structured around what students are expected to be able to do at the end—individually isolate caffeine from a consumer product as a culminating lab practical, using IMFs to justify solvent choices and determining procedural details. We have found that instructors can select a challenging multilevel experiment that incorporates the application of IMFs in multiple ways and backward design the course so that students are able to complete this experiment individually and autonomously at the end of the semester. By incorporating evidence-based pedagogies to foster meaningful learning and repetition of techniques and IMF concepts in different contexts, we promoted opportunities to learn from mistakes and prioritized student decision making. This approach involved faculty collaboration and spanned several semesters of iteration. In our experience, a cumulative lab practical motivates students to learn the techniques and take responsibility for learning. We propose that the backward design process with a central theme, such as the application of IMFs in our case, is especially well suited to planning a chemistry laboratory course. However, even with an entire laboratory course centered around applications of this critical concept, we discovered there were still gaps in students’ abilities to apply IMFs.

## Introduction

Recent research has emphasized the importance of incorporating engaging approaches in laboratory education that promote discovery-based and cooperative learning.^1−3^ It is important to structure the curriculum in a way that builds upon students’ prior knowledge, provides opportunities for repetition, and supports them in tackling new challenges. Creating an environment that encourages motivation for laboratory work and enhances student confidence is essential, as meaningful laboratory learning takes place at the intersection of the psychomotor, cognitive, and affective domains of learning.^4−6^ Implementing such approaches requires intentionally developed laboratory courses.^7−11^

**Figure 1:**
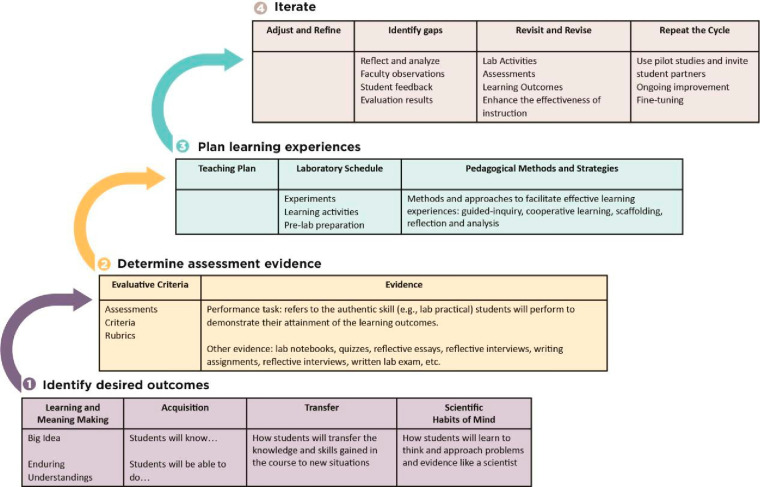
Backward design of the Principles of Reactivity laboratory course followed a four stage process.

Backward design is a curriculum development model in which instructors start the design of a course by determining the desired result—what they would like the students to know and be able to do at the end of the course (Figure 1).^12,13^ Using backward design to develop a laboratory course requires prioritizing the intended learning, going beyond generating a list of experiments or covering a broad range of topics and activities without a unifying purpose. Many excellent examples of backward design of laboratory courses have been published.^10,14−19^ Herein, we discuss the backward design of a laboratory course where intermolecular forces (IMFs) were incorporated as a central concept, with an individual cumulative lab practical that requires students to make choices that should be dictated by a meaningful application of IMFs. While IMFs are typically covered in a single lecture session, applying them in various contexts and avoiding misconceptions can be quite challenging.^20,21^ Learners must be able to make sense of molecular structure and transition from perceiving molecules as discrete entities to recognizing them as dynamic units that interact with one another. While an entire laboratory course centered around a meaningful application of IMFs is a novel approach, we discovered that there were still gaps in students’ abilities to apply these foundational concepts.

## Context

Oxford College, a small liberal arts-intensive division of Emory University, enrolls about 1000 students for the first two years of their undergraduate education. Oxford’s general education program emphasizes inquiry-based learning, and science courses enroll a maximum of 24 students with faculty teaching both lecture and laboratory. Students spend their final years on Emory’s Atlanta campus, benefiting from the resources of a research university. In 2017, Emory and Oxford introduced “Chemistry Unbound”, an innovative four-year undergraduate degree program based on enduring thematic frameworks built around core ideas and scientific practices.^22^ To highlight the importance of the laboratory in learning chemistry, the laboratory courses were developed as independent two-credit hour courses. The separation of lecture and lab presented a chance to rethink the entire laboratory curriculum. The first lecture course in the Chemistry Unbound sequence (Structure and Properties) emphasizes chemical structure and its connection to physical properties, culminating in a 35 minute introduction to IMFs followed by active learning using models. Principles of Reactivity Laboratory, the second laboratory course and the focus of this article, replaces General Chemistry II lab. Our aim was to design a lab course that picks up where the Structure and Properties lecture course ends; building on these concepts and acting as a bridge to the third lab, which focuses on organic synthesis. We also wanted students to complete the lab prepared for undergraduate research experiences. Instead of starting with a list of topics or experiments, we employed backward design centered around application of IMFs, which proved to be an excellent tool for narrowing our focus; emphasizing depth over breadth.

When designing a course using backward design, instructors must prioritize the desired learning outcomes and evidence of student understanding. Activities should revolve around Big Ideas to foster connections and deeper comprehension, promoting meaningful application of knowledge (see Box 1). Scaffolded learning experiences guide students to progressively explore and grasp the complexity of these ideas.^16−19,23^ This approach ensures that every task and instructional practice serves a purpose and aligns with the overall learning goals. Although challenging, eliminating less relevant content and activities can enhance the overall effectiveness of the curriculum.

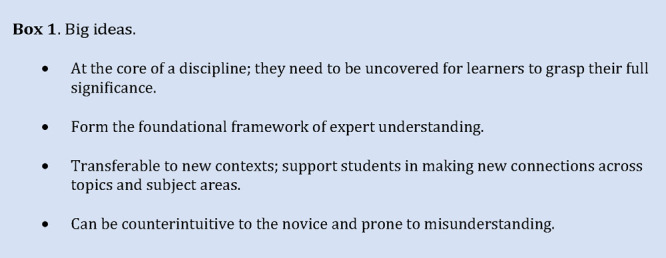



## Stage 1: Identifying the Desired Outcomes

Prior to implementing the curriculum change, applications of IMFs were traditionally taught in the first semester organic laboratory. Instructors observed that students encountered difficulties in applying this seemingly simple concept. While students grasped polar, nonpolar, and “like-dissolves-like” they struggled with translating knowledge of IMFs in sophisticated applications. Our course design aims to provide students with a more meaningful foundation of IMFs to build upon in subsequent courses and an ability to transfer their knowledge to diverse and novel situations.

The process began by selecting a traditional experiment exemplifying the thinking skills and techniques students should demonstrate at the end of the course. We wanted students to demonstrate performance of targeted lab skills while using molecular structures and IMFs to make choices and procedural decisions. The isolation of caffeine from a tea bag is a well-known multistep experiment which met these goals and is interesting to students.^24−27^ Another goal was to motivate students to care about learning the techniques and concepts and to gain confidence.^28^

The desired learning outcomes for Principles of Reactivity Lab are for students ***to design, execute, and justify their own procedure to isolate caffeine from a consumer product***. They are provided a mock consumer product with the chemical structures of four or five ingredients. Students communicate their procedure using a flow scheme which also provides scaffolding that aids students in visualizing the separation process on the symbolic level. Several concepts were identified from the associated lecture course that relate to this experiment; other concepts are specific to the laboratory (Table 1).

**Table 1:** Necessary Skills and Knowledge for the Laboratory Practical

*Techniques*	*Concepts*
*Liquid–liquid extraction*	IMFs, solubility, thermodynamics, equilibrium, partition coefficients
*Acid–base extraction*	Acid–base reactions, functional groups, resonance, changing IMFs, ion-dipole forces
*Washing/drying/decanting*	Drying agents, hydrates, solubility, ion dipole forces
*Rotary-Film Evaporation*	IMFs and phase changes, vapor pressure, boiling point, evaporation/condensation, reduced pressure
*Thin-Layer Chromatography*	Mobile and stationary phases, partitioning, elution, R_f_ values, IMFs and separation based on polarity and H-bonding ability
*Melting Point Determination*	IMFs, melting point depression, entropy of phase changes
*Use of Analytical Balance*	Law of Conservation of Mass

What mattered to us was that students understood the concepts well enough to *make their own choices* during the practical and be well-prepared for a research experience. Backward design made it easy to articulate course learning outcomes in terms of process skills and conceptual understandings (Table 2).

**Table 2:** Student Learning Outcomes for *Principles of Reactivity Lab*^a^

Category	*Students should demonstrate the ability to*:
**Process Skills**	• *Perform* a liquid–liquid extraction in a separatory funnel
	• *Identify* the phases in a separatory funnel
	• *Label* containers
	• *Perform* sequential extractions, keeping track of which layer goes back into the separatory funnel
	• *Use* drying agents meaningfully
	• *Measure* mass of round-bottom flask prior to rotary evaporation
	• *Execute* meaningful TLC (*spot* samples, *develop* the plate, *visualize* the spots)
	• *Determine* a melting point
	• *Perform* techniques independently
	• *Follow* lab safety protocols
	• *Practice* good time management
	• *Recover* sufficient product for analysis
**Design Choices**	• *Choose* a suitable organic solvent for liquid–liquid extraction, given molecular structures of components of a mixture and a limited set of solvent choices
	• *Choose* an appropriate aqueous phase for acid–base extraction, given molecular structures of components of a mixture and a limited set of aqueous solutions
	• *Choose* an appropriate mobile phase for TLC (based on previous experiments)
	• *Choose* an effective number of extractions
	• *Determine* which layer is top/bottom and which to put back into the separatory funnel
	• *Choose* which standards to include on the TLC plate
	• *Design* a TLC plate choosing appropriate controls and replicates
	• *Design* a melting point determination and *choose* an appropriate number of replicates
	• *Record* melting points as ranges
**Predictions**	• *Predict* the preferential solubility of compounds between two immiscible phases
	• *Identify* any acidic or basic functional groups, if present
	• *Draw* structures of conjugate acids and bases
**Justifications/Explanations**	• *Justify* claims using chemical structures and properties (e.g., polarity) and IMFs
	• *Connect* visual observations to molecular or representational levels
	• *Explain* how preferential solubility of a compound can be changed by altering its structure, properties, and predominant IMFs
	• *Explain* the solubility of charged species by meaningful use of ion-dipole forces
	• *Justify* the performance of multiple sequential extractions as opposed to fewer, larger-volume extractions
	• *Analyze* TLC results using meaningful reasoning
	• *Analyze* melting point data using meaningful reasoning
	• *Make appropriate claim(s)* about the success of a separation process, supported by quantitative (yield, melting point) and qualitative (TLC, procedural observations) evidence

a The learning outcomes for the course were identified by the faculty after breaking down all parts of the culminating experiment. The lab practical is meant to serve as an authentic assessment of student learning.

## Stage 2: Deciding What Evidence of Learning Will Look Like

One of the most appealing aspects of using backward design for laboratory courses is the opportunity to identify how students will demonstrate their learning. In our context, we selected a laboratory experiment that pulls together many aspects of a chemistry lab experience, applies IMFs in multiple contexts, bridges concepts between general and organic chemistry laboratories, and connects to students’ lives. This experiment became the culminating experience for the semester, streamlining the content and learning outcomes for the course (see Box 2). It was assumed that if students could *independently conduct the experiment, design the flow scheme* and *justify their choices*, it would *directly indicate their achievement of the course learning goals*.
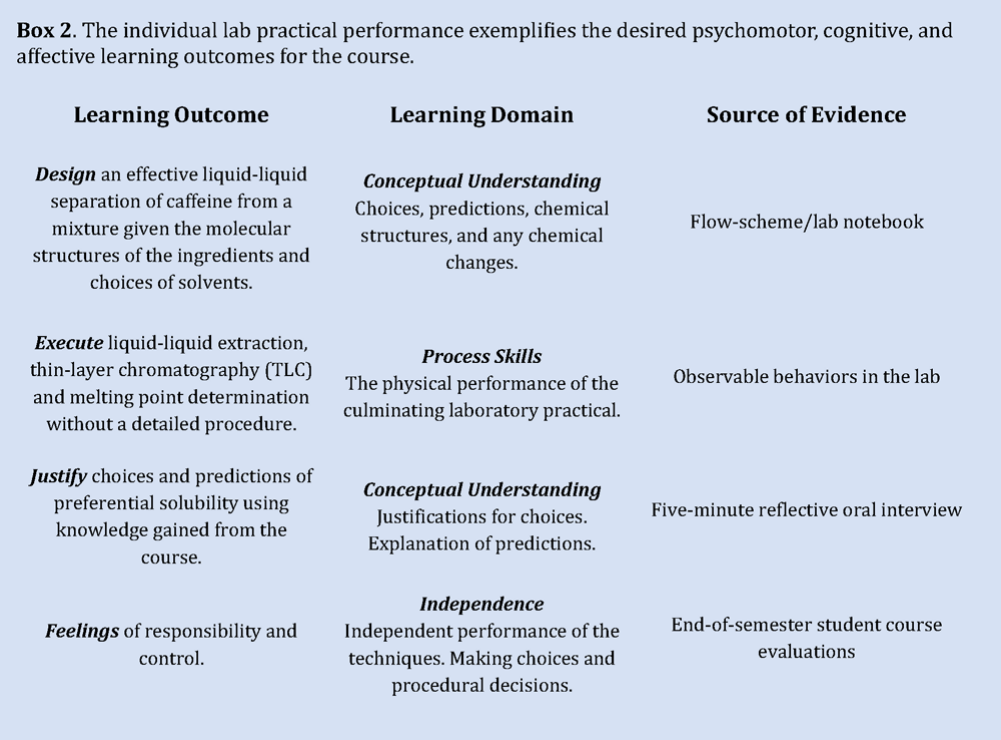


The process involved identifying the expert thinking required for the culminating experiment and modeling that thinking for novice learners. Mastering IMFs is vital for students’ understanding of chemistry, as IMFs offer a framework for explaining phenomena such as states of matter, phase transitions, solubility, chromatography, and more. For experts, IMFs serve as a central, organizing principle that connects and provides coherence to various techniques in the chemistry laboratory and has wide-ranging implications for understanding the properties and behavior of molecules and materials. Creating a laboratory course centered around the application of IMFs also allowed for the inclusion of experiences involving complexity and nuance, fostering an appreciation for the inherent uncertainties and limitations of scientific knowledge.

## Stage 3: Planning the Learning Experiences

Finally, the laboratory schedule was planned, and we selected experiments (Table 3). Liquid–liquid extractions can be challenging for students because solubility and partitioning depend on many factors including solvent polarity, predominant IMFs, chemical structure, thermodynamics, and equilibrium. The incorporation of an acid–base reaction to change the structure, predominant IMFs, and solubility of a component increases the complexity. The plan was to structure the course carefully to support students in developing their understanding over time, requiring students to transfer their knowledge from one lab session to another. Evidence-based pedagogies were incorporated to promote meaningful learning, with a deliberate focus on providing opportunities for concept and technique repetition across various contexts. Studies indicate that students recognize the value of repetition in lab courses as it aids in the development of their technical skills and content knowledge, aligning with the principles of deliberate practice.^29^ The “practice” lab practical, where students are allowed to work in pairs and are encouraged to ask questions is meant to consolidate student learning and was designed with desirable difficulties. The consolidation of learning is important for student success on the lab practical itself and instructors provide support through targeted discussions with each group and by feedback on individual flow schemes.

**Table 3:** Principles of Reactivity Laboratory Schedule F2021–S2022

Week	Experiment	New Skill(s)	Past Skill(s)/Practice
**1**	**IMF “Workshop”**	Johnstone’s Triangle for chemistry; representation of IMFs	IMFs (Structure and Properties course)
**2**	**TLC Analysis of an OTC Pill**	TLC	IMFs
**3**	**Are these solids the same substance?**(28)	Melting point	TLC, chemistry triangle, IMFs
**4**	**Predicting the Solubility of Chemotherapy Drugs**(29)	Liquid–liquid partitioning; predicting solubility based on structural features toolkit (predominant IMFs, H-bond donors/acceptors and thermodynamic considerations)	Chemistry triangle, IMFs, identifying and drawing permanent bond dipoles, representation of IMFs
**5**	**Liquid–Liquid Extraction and the *K*_D_ of Caffeine**	Separatory funnel, drying agents, rotary evaporation, equilibrium in a new context	IMFs, TLC, predicting solubility toolkit
**6**	**Colorful Liquid–Liquid Extraction**(30)	Multiple extractions acid–base extraction drawing flow schemes	IMFs, predicting solubility toolkit, separatory funnel
**7**	**Practice Lab Practical**(30)**(in pairs)**	Washing with brine	IMFs, TLC, melting point, predicting solubility toolkit, separatory funnel, drying agents, rotary evaporation, multiple extractions, acid–base extraction, drawing flow schemes
**8**–**9**	**Independent Lab Practical**	(No new skills)	IMFs, TLC, melting point, predicting solubility toolkit, separatory funnel, drying agents, rotary evaporation, multiple extractions, acid–base extraction, drawing flow schemes
**10**	**In-Person Quiz**	(No new skills)	Representing IMFs, predicting solubility toolkit, chemistry triangle

### Structural Features and Strategies to Enable Independent Decision Making

**Figure 2:**
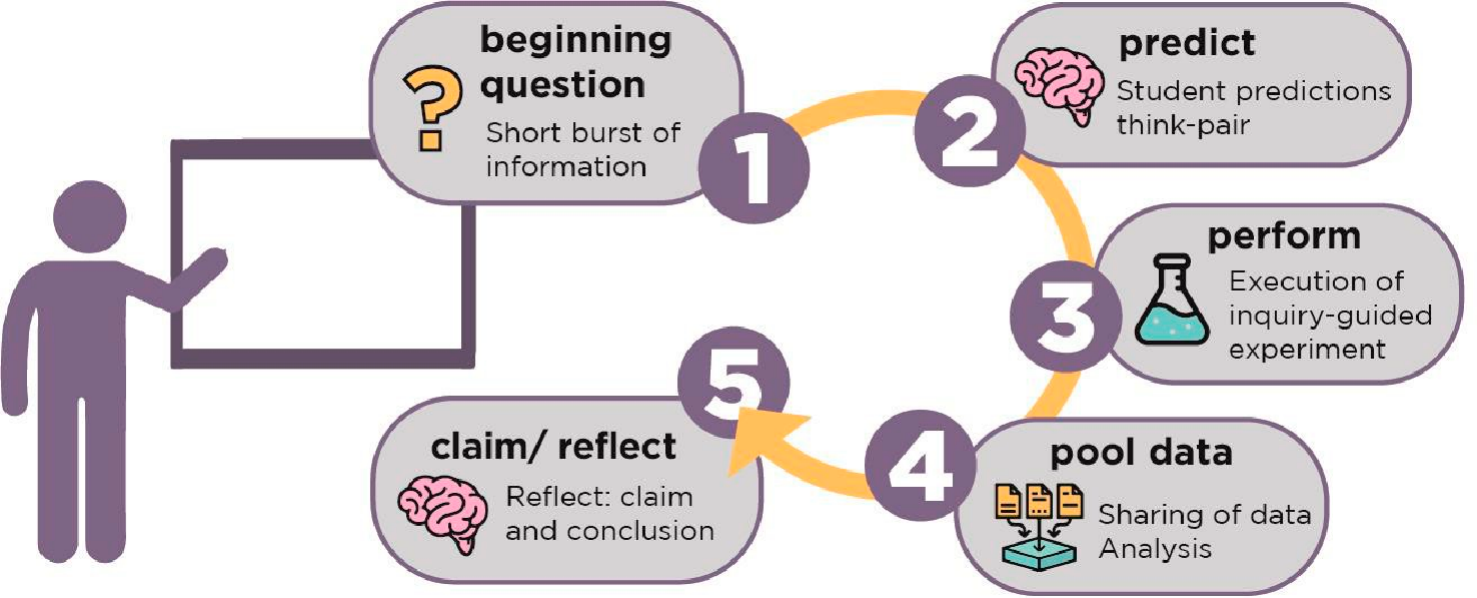
Orchestrated laboratory experience.

During the first few iterations of the course, the faculty developed an orchestrated laboratory experience (Figure 2) that is generally repeated each week.^33^ The students read background information on a new technique and a lab handout with a Beginning Question (BQ) and skeleton procedure before coming to lab. Teams of four students share lab benches and fume hoods, with a projector screen at the front of the lab. The instructor guides the lab sessions using slides, providing interactive materials on underlying concepts, continuously building on knowledge of IMFs.

Students write predictions and interact with their team to identify variables and make procedural decisions. Lab partners work together to collect data and report it on a class spreadsheet. Teams analyze the pooled data and are supported in finding patterns, trends, and making claims in their notebooks. Throughout the process of collecting data the students are guided through overarching takeaways using the slides. If students’ original predictions are incorrect, they are congratulated for engaging in science and are tasked with explaining how their thinking changed based on the experimental evidence.

Our approach to encouraging meaningful learning in the lab incorporates recommendations specific to chemistry laboratory education reported in the literature.^4,6,34−37^ By working toward a practical that combines all taught concepts and techniques, course learning goals align with student affective goals, motivating engagement and responsibility. Cognitive goals are emphasized through faculty orchestrating the laboratory experience and encouraging meaning making, ensuring active participation from each student, removing any opportunities to leave the lab session early, using the same five solvents every week to ensure they are familiar to students, and eliminating procedures that encourage division of labor.

Mistakes are expected as part of the learning process and are promoted as valuable learning opportunities. By providing procedural choices throughout the semester that lead to data failure, students analyze larger pooled data sets and consider methodological flaws.^38^ Several experiments have been modified to include substances that take advantage of common misconceptions.^39^ Students often make predictions based on misconceptions that are brought to light through experimentation. Embracing data that requires a change in mental models is a vital aspect of students’ growth as budding scientists.

Rather than directly answering questions, faculty support students’ growing independence by guiding students to find their own answers. Instructors new to inquiry teaching struggle with not providing direct answers, feeling that answering questions is the best way to be helpful. However, guided-inquiry methods have been shown to improve student affect, perception of learning, and laboratory competence.^40−43^ Inquiry instructors clarify and probe student questions to determine needed support without revealing answers. They redirect questions to prompt critical thinking or integration of concepts. Collaboration and discussions are encouraged; often the instructor will step away, returning to monitor independent discoveries. This relies on an environment where students can comfortably ask questions and share thoughts. Instructors celebrate critical thinking, discussions, and “aha” moments. In our experience, cultivating these “aha” moments is important for improving understanding of IMFs.

## Stage 4: An Iterative Process

Helping students achieve the outcomes involved collaboration among the faculty. During the multiple iterations of this course, the faculty met regularly and identified areas where more intentional and explicit scaffolding was needed to build connections between core concepts, the key representations of those concepts, and the contexts and practices in the lab.^44^ The authors came together with a wide variety of teaching experiences. Two senior faculty had experience with inquiry-based teaching and had implemented a course-based undergraduate research experience (CURE) using backward design. Most junior faculty were recent hires; all were experienced teachers but were less experienced with inquiry-based laboratories.

There were multiple semesters of careful observation, assessment, and iteration, starting from a place where everyone felt comfortable. Experiments published in this journal were adopted;^30−32^ over time they were modified to better scaffold student learning in our context. We moved slowly and incorporated multiple perspectives and strengths into the course design. During the first few semesters, faculty met weekly to discuss teaching challenges and successes, held workshops, and formed a teaching circle to explore scholarly approaches to address challenges. The use of orchestrated slides played a significant role in our development process. The integration of these slides facilitated a streamlined approach and uniformity in student experience. As successful teaching techniques were discovered, they were integrated into the slides, eventually becoming the standard.

To illustrate our process, the following section focuses on the iterative development of one experiment. A published exercise was adopted that consists of a series of “mini-experiments” comparing the solubility of structurally similar compounds; students record their observations.^31^ For instance, one mini-experiment consists of observing the solubility of a series of alcohols in water. From each mini-experiment, it was hoped that students would gain insight into specific structural features that influence solubility. The exercise was modified to provide a BQ for each mini-experiment. Students made individual predictions, identified variables, performed experiments in groups, pooled class data, and developed claims to address the BQs. Assessment of the postlab writing assignment indicated that, in our context, students were not learning the important concepts. Using a *students-as-partners* approach, a student intern contributed to the revision of laboratories following the initial implementation.^45^ Recognizing the significance of the solubility lab for student success, concerted effort was directed toward it. Aspects of the lab were reviewed with the intern, and the instructor explained how sense could be made of each mini-experiment and results. The student said a remarkable thing (Box 3) that changed our perspective on designing laboratory activities.
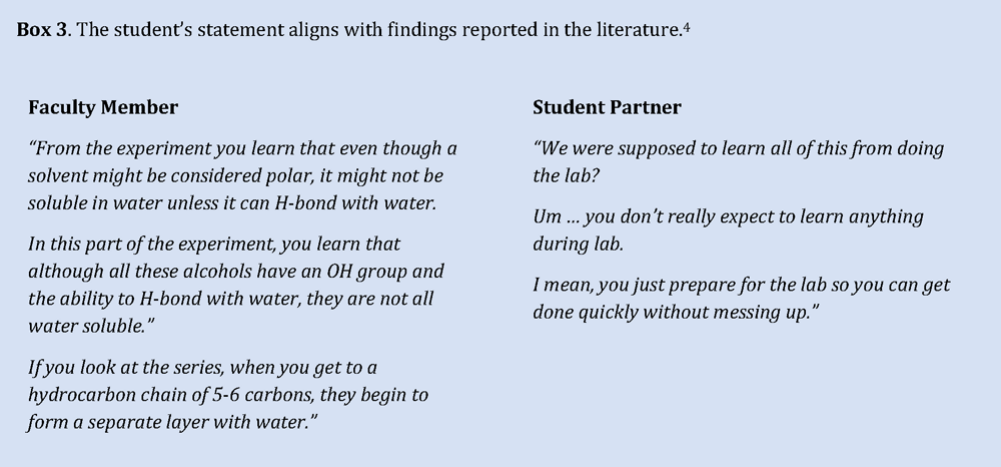


The experiment was transformed into “Predicting the Solubility of Chemotherapy Drugs”, challenging students to focus on learning solubility concepts and a more nuanced application of IMFs *during the lab session*. The overarching BQ requires predicting water solubility of an individually assigned chemotherapy drug based on its molecular structure at the end of the lab session; students are also asked to justify their prediction using evidence from the experiments. This change kept students engaged, prompting questions and connections to their existing chemistry knowledge. The approach also introduced the concept of predominant IMFs and challenged students to deal with ambiguity in a first-year laboratory course. Key takeaways from the mini-experiments are elicited from students, but there is always a follow-up on the orchestrated slides to make sure that everyone is on the same page.

During the second implementation of this approach, one faculty member asked students to discuss their mental models for predicting solubility, tallying their responses on the whiteboard (“like dissolves like,” “hydrogen bonding,” etc.). During the mini experiments, as some of their mental models were discovered to lead to incorrect predictions, the instructor would cross them out. This turned out to be a terrific way to help students focus on adapting their mental models to accommodate new evidence. This approach is known as the MORE Thinking Frame^46^ and encourages students to consider their mental models and reflect on how their models align with experimental evidence. This metacognitive approach simulates the thought processes of practicing scientists.

### The Lab Practical

The lab practical was designed as a culminating experience for students to demonstrate both process and conceptual skills. Scaffolding fosters success by ensuring that students have opportunities to repeat and reinforce their technical and thinking skills. While a lab practical can be a stressful experience, the goal was to create a positive environment that emphasizes learning over perfection. It is rewarding to observe that the lab practical serves not only as an assessment but also as a learning experience, enabling students to solidify their understanding while gaining confidence and developing their identity as scientists. The lab practical contributes 20% to the overall course grade, which motivates students to focus on mastering laboratory concepts and techniques throughout the semester.

At Oxford, laboratory sessions are 2 hr and 45 minutes. To accommodate individual assessments, the lab practical tasks were originally conducted over four lab sessions, with 12 students completing half of the assessment per week. Students share fume hoods with dividers to promote individual and focused work. Students are presented with their mock consumer product at the beginning of the practical session; different mixtures are used for each lab section to ensure variety and fairness. While most laboratory sessions have a highly organized structure involving predictions, data collection, analysis, and class discussion, the lab practical session is much more open-ended. At the beginning of the lab session, students are reminded that they may not talk to one another during the lab practical and are provided with encouragement (see Supporting Information). Since Fall 2021, students have one lab period to complete the isolation and analysis of caffeine. They are not permitted to use lab notebooks, which allows assessment of their abilities without written prompts. Large sheets of paper are provided with the molecular structures of their given mixture, where they record their flow schemes, procedures, and data. The cumulative practical is the only experiment during this laboratory course where some students finish significantly faster than others since everyone is working at their own pace. To remove time pressure, we give all students the entire lab session for the practical, which is more time than is necessary. This may be beneficial for students with organizational difficulties, who struggle with systematically working and recording the flow scheme and results.^49^

The primary components of the composite lab practical score are the process and conceptual scores. The process score assesses students’ ability to perform specific tasks, such as using the separatory funnel, drying agent, and TLC. The conceptual score evaluates their ability to apply course concepts to procedural choices. Conceptual mastery is assessed using the flow scheme and a 5 minute reflective oral assessment; the latter gives students a chance to explain the thinking behind their procedure. Although an oral assessment with an instructor can be a stressful experience, it provides an invaluable opportunity to probe student thinking and is significantly faster to assess than pages of written work. Since some students find even simple, on-the-spot questions to be challenging and may require more time to prepare responses, we acknowledge that this may pose challenges for some students.^47^ While we do allow wait times for responses between 3 and 5 minutes, future iterations will incorporate multiple opportunities for students to provide their justifications.

Full points for time management are earned by completing the experiment and turning in the flow scheme within the generously allotted time. Full points for independence come from performing the experiment without asking questions about previously reinforced techniques. Assistance is available for unusual problems (like emulsions) as well as techniques that were not major foci of the course.

**Figure 3:**
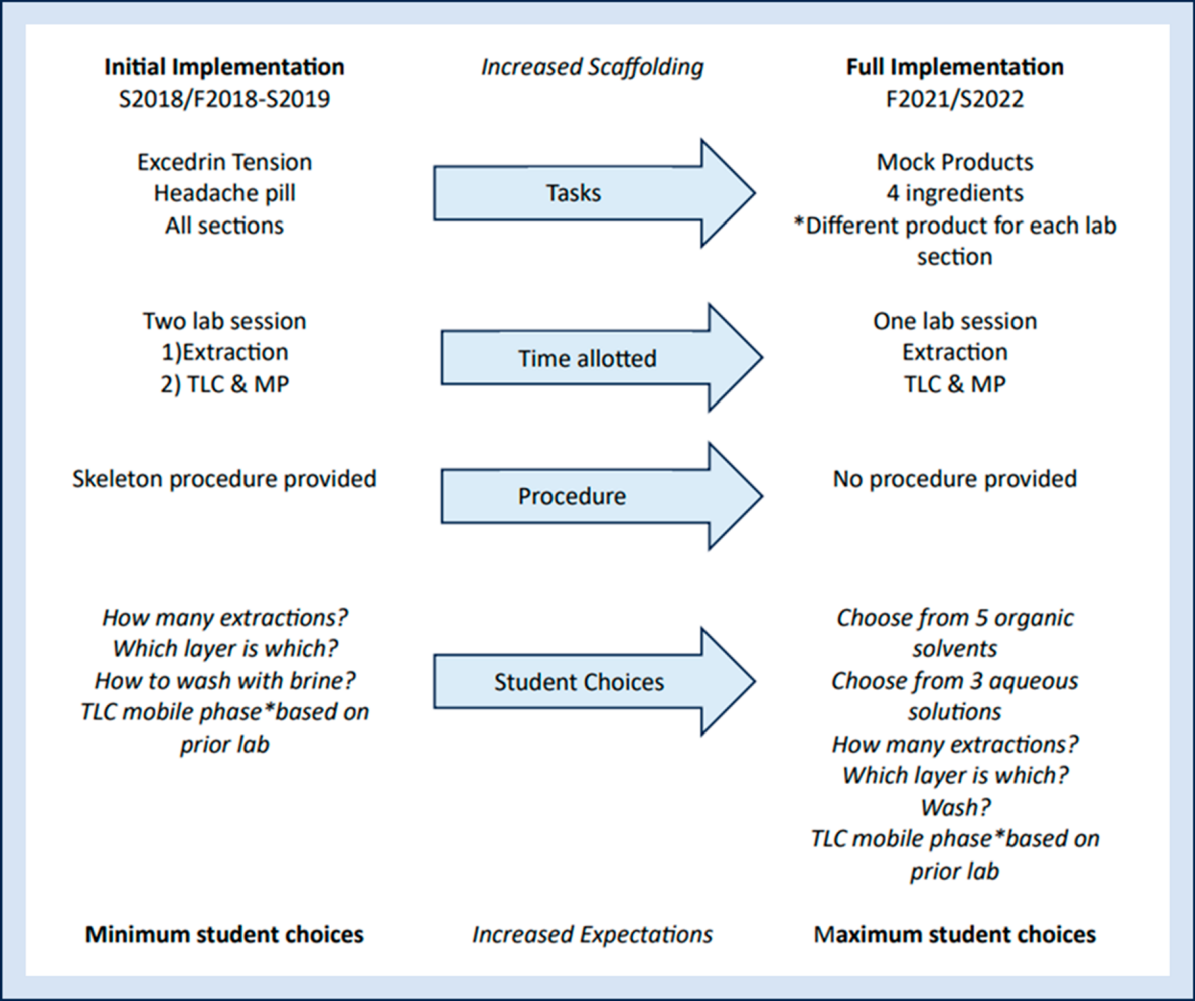
Evolution of the lab practical over time

For initial attempts at implementing the practical, students were provided with a BQ and a basic procedural outline. They were given general guidance on how to approach the procedure and made limited choices for isolating caffeine from the same consumer product (Figure 3). After the first implementation, the faculty met to discuss observations. One of the most important things we agreed on is that while the lab practical is a summative assessment, it would be prioritized as a learning experience. If students were struggling to complete basic laboratory tasks, it was obvious even to them. The prime objective was for all the students to leave the lab after the practical feeling they had learned something about themselves. Some students learned to be more confident, as they were able work more independently than they had ever anticipated; other students discovered that they needed to work harder to be successful.

As opportunities for further developing students’ techniques were identified through observation and discussion, more scaffolding was incorporated such as reflective writing assignments that helped students enhance their conceptual understanding. As thinking skills showed improvement, instructors became more comfortable with implementing the lab practical, which led to the current version with increased expectations, a greater emphasis on individual decision-making by students, and a significantly shorter time allotment of one lab session.

## Evidence of Student Growth

*“To understand is to have done it in the right way, often reflected in being able to explain why a particular skill, approach, or body of knowledge is or is not appropriate in a particular situation.”* Wiggins & McTighe (2005)^12^

To gauge students’ progress toward course objectives, faculty collaborated on the development of a process checklist used to assess students during the lab practical (see the Supporting Information). As we finalized the details of the process checklist, we agreed that expert performance of the techniques was not reasonable nor expected from second semester chemistry students. Through discussion, the faculty negotiated what would count as evidence that students had demonstrated each process skill. Each instructor was supported by a TA who also used the process checklist; having a second observer helped mitigate the difficulties associated with evaluating 12 students at one time. To promote fidelity across multiple sections, the lab director attended the first hour of each lab practical, supporting instructors and TAs as they made observations. We found it was useful to identify what it looks like when a learner has an incomplete understanding. In practice, it is much easier to scan the room for anomalies than to watch each student for a perfect performance.^48^ Students were required to interact with the instructor after using the drying agent and after performing TLC. During these interactions, the instructor observed the results of student work and asked clarifying questions.^49^ The percentages of students who demonstrated individual process outcomes by observable behaviors during the lab practical are shown in Table 4.

**Table 4:** Evaluation of Process Outcomes, Fall 2021–Spring 2022 (*N* = 102)

Students should demonstrate the ability to	Percentage of students who demonstrated outcomes by observable behaviors or flow scheme
*Execute* the **liquid–liquid extraction process** without a detailed procedure:	
• perform liquid–liquid extraction using a sep funnel (burping, shaking, and holding the lid on)	**88**
• perform sequential extractions, keeping track of which layer goes back into the sep funnel	**91**
• wash the correct phase	**74**
• use drying agent meaningfully	**70**
• recover sufficient caffeine for TLC/mp	**81**
*Execute* the **TLC process** without a procedure	**91**
Determine a **melting-point**	**93**
*Other considerations*:	
• follow lab safety protocols without reminders	**96**
• perform techniques independently	**94**
• practice effective time-management	**89**

During the initial implementation in Spring 2018, more than half of the students struggled with the faculty expectations for handling the separatory funnel and using drying agents. At that time, 15% of students did not meet the competency threshold, which was defined as independently isolating enough caffeine to perform a TLC analysis. To address these issues, modifications were introduced in the initial, repetition, and practice lab practical experiences. We began to set the tone for the semester by explaining what students would be expected to do at the course’s end. We found it helpful to remind students (weekly) that they will need to perform the lab techniques independently at the end of the semester and demonstrate their understanding of course concepts. Since that time, the percentage of students who have not met the competency threshold has not exceeded 3%.

Conceptual understanding was assessed through student design choices, solubility predictions, and justifications (see Table 5 and 6). Assessments included observing and interacting with students during the lab practical,^48^ reviewing flow schemes, and conducting 5 minute reflective oral assessments (see Supporting Information). The oral assessments were recorded for later analysis using an instructor-facing rubric that was developed and refined over time (Table 7). For the purpose of writing this manuscript, and to ensure reliability and consistency in assessment, two faculty members listened to each oral assessment recording together, scoring them in conjunction with the corresponding flow scheme. The rubric provided a structured framework for evaluating the quality and depth of students’ responses, considering the content of their responses and alignment with their flow scheme. Students were given feedback on process, conceptual understanding, and independence using a different, student facing rubric that is more holistic in nature (see the Supporting Information).

**Table 5:** Evaluation of Conceptual Outcomes for the Liquid–Liquid Extraction F2021–S2022 (*N* = 102)

Based on the molecular structures of the compounds in the mixture, students should demonstrate the ability to	% of students who demonstrated outcomes
*Choose* an appropriate organic phase for liquid–liquid extraction	**89**
*Choose* an appropriate aqueous phase for acid–base extraction	**81**
*Predict* the molecular consequences of aqueous phase choice (acid–base reaction)	**61**
*Predict* the preferential solubility of all substances between the two immiscible phases	**57**
*Choose* an effective number of extractions	**79**
*Justify* the performance of multiple sequential extractions as opposed to one larger volume extraction	82

**Table 6:** Evaluation of Conceptual Outcomes by Justifications and Explanations F2021–S2022 (*N* = 102)^a^

Composite Justification and Explanation Rating	Percentage of Students (*n* = 102)
Exemplary	14%
Competent	36%
Emerging	27%
Novice	23%

a Data were collected from triangulation of flow schemes and 5 minute oral assessments using the instructor-facing conceptual rubric. Two faculty listened to recordings of all oral assessments together and agreed upon a composite score for each student.

**Table 7:** Instructor Facing Rubric for Conceptual Understanding

Exemplary	Competent	Emerging	Novice
*Solvent* choices *make sense for the given mixture.*	*Solvent* choices *make sense for the given mixture.*	*Solvent choices make sense for the given mixture.*	*One or more solvent* choices *do not make sense for the given mixture.*
*Justifications for solvent choices include use of molecular structural cues without prompting.*	*Justifications for solvent choices include use of molecular structural cues after prompting.*	*Justifications may include some structural features after prompting but are not clearly connected to choices.*	*Justification may not include any mention of structural features, even after prompting.*
*Justification for solvent choices includes effective language for IMFs without prompting.*	*Justification for solvent choices may include effective language for IMFs after prompting.*	*Justification for solvent choices may include language for IMFs after prompting but the language choices may not be effective.*	*Narrative may include random use of IMF jargon (polarity) with no clear connection to choices.*
*Solubility predictions make sense for the molecular structures and extraction system.*	*Most solubility predictions make sense for the molecular structures and extraction system.*	*Some solubility predictions make sense for the molecular structures and extractions system.*	*Predictions of solubility do not make sense for the molecular structures.*
*Explanations are clear that preferential solubility was changed by altering structure, properties, and predominant IMFs.*	*Explanations of acid–base reactions are meaningful, but do not explicitly include the need to use acid–base reaction to change solubility.*	*May seem lost in trying to identify and explain the details of acid–base reactions. Explanations not clear that water is the solvent.*	*Explanations do not mention acid–base.*
*May demonstrate integration of conceptual frameworks (for example: IMFs and acid–base).*	*May demonstrate disjointed conceptual frameworks*	*No clear demonstration of either conceptual framework.*	*May not be able to identify aqueous or organic layers.*
			*No use of either conceptual framework*

The lab practical might provide meaningful assessment of student learning, but some students seemed to mimic satisfactory performance without a deep understanding. For example, most students were able to choose solvents and aqueous solutions that allowed them to isolate caffeine, however, they were not always able to justify why those choices were successful. This is where we have found that a five-minute reflective oral assessment provided more insight than written answers. Some students seemed able to parrot the language required to explain phenomena, but their explanations fell apart upon probing while some students were able to improve their answers with a small amount of prompting. It is also important to consider that students who have not fully grasped acid–base concepts in the lecture course will struggle to apply them effectively in the lab. Valuable insights into student challenges and misconceptions were gained from the transcripts of the five-minute reflective oral assessments. As expected, many students revealed misconceptions, and even some students rated as Competent displayed surprising conceptual gaps. Some interviews revealed various misconceptions regarding the term “aqueous phase” (see Box 4). Targeted interventions have been implemented to address these issues in the initial, repetition, and practice lab practical experiences.

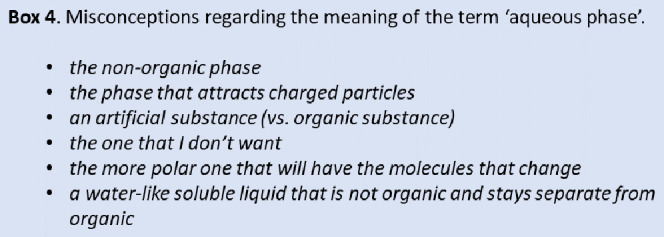



Our focus on teaching students to apply a more meaningful understanding of IMFs has required that we spend significant time teasing out misconceptions; many activities were incorporated into the course to target representational competency and build mental models. However, there are still many gaps in student learning even after an entire semester focused on this Big Idea. There is quite a difference between how quickly IMFs are covered in a typical lecture course versus how long it takes to practically master these concepts. By teaching in this focused way, we have observed a complex array of difficulties that learners experience as they work toward an understanding of IMFs. This work will be presented in a future manuscript.

### Affective Themes

Collaborative thematic analysis of unprompted student comments was performed by two faculty on anonymous routine end-of-semester course evaluations (IDEA forms) yielding qualitative insights into the affective domain. The themes suggest that the Principles of Reactivity Laboratory course is effective in bridging the cognitive, affective, and psychomotor domains of learning for many students.

#### 1 Embracing Mistakes

Strategic efforts have promoted a positive attitude toward making mistakes in the lab (Box 5). Embracing mistakes as learning opportunities is essential for developing scientific inquiry skills. By acknowledging mistakes, reflecting on them, and cultivating a growth mindset, students engage in critical thinking and improve their understanding of concepts, bridging the cognitive and affective domains.

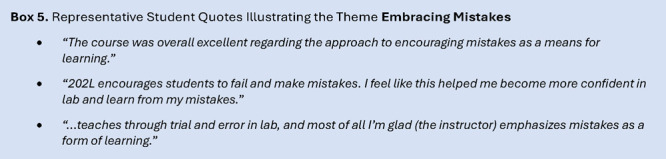



#### 2 Focus on Understanding

Students’ positive emotions and attitudes toward critical thinking and independent learning may have led to increased self-efficacy, a sense of accomplishment, and a willingness to embrace challenges, all of which are essential affective factors that can enhance their overall learning experience (Box 6).

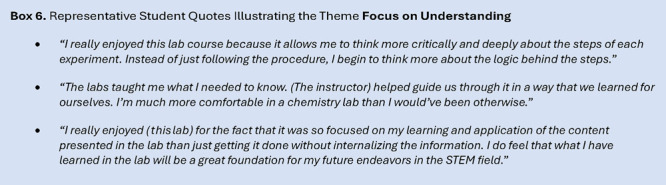



#### 3 Fostering Independence

Students felt empowered and confident in their ability to tackle complex problems and make informed decisions (Box 7). These beliefs and feelings contribute to the affective domain by influencing students’ motivation, engagement, and emotional responses to the learning process.

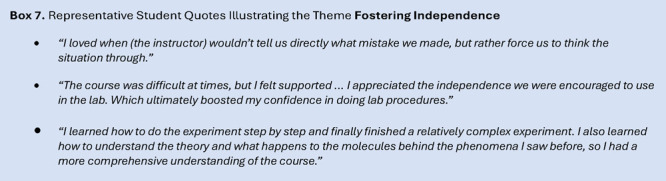



Example comments from multiple individuals on unprompted end-of-semester evaluation forms (IDEA).

## Limitations

We shifted our evaluation of students’ conceptual understanding from written questions to an oral reflective assessment to add flexibility to the assessment. However, the use of oral interviews suffers from the drawback of working against inclusive practices as they can be challenging for some neurodivergent students.^47^ We have started experimenting with offering alternative modes for students to communicate their conceptual mastery.

Using the same lab practical each semester for a multisection laboratory course provides an opportunity for students to learn about the assessment from their peers before completing it in the lab. To maintain the integrity of the assessment, we vary the consumer product we provide students in each lab section and give different options for organic solvents. We also have a bank of reflective oral assessment questions, giving instructors the ability to further probe students’ understanding when it appears that they may have memorized explanations.

We have been through several iterations of the student-facing rubric with the goal of providing constructive feedback without sharing specific details on how to succeed in the lab practical. In one iteration of the rubric, we used the terms *exemplary*, *competent*, *emerging*, and *novice* to describe students’ progress toward mastering process and conceptual skills. However, we discovered that this language made some of them feel bad about their performance. We have since adjusted our wording on the student facing rubric to *well demonstrated*, *demonstrated with a few issues*, and *not well demonstrated*—language which clearly describes the evaluation of specific skills and not the student.

## Conclusion

Our original assumption was that if students could *independently conduct the experiment*, *design the flow scheme*, and *justify their choices*, it would *directly indicate their achievement of the course learning goals*. 97% of our students were able to independently conduct the experiment and design the flow scheme. The course design is most effective at promoting students’ acquisition of techniques and developing positive attitudes toward working in the lab. Learner provided justifications for their choices gave insights into how well students could apply IMF concepts in what we hoped would be an authentic assessment. While 50% of students justified their choices using molecular structural cues and IMFs language, it was not possible in this context to disentangle IMFs from other conceptual frameworks such as acid–base or equilibrium.

We have shown that backward design of a laboratory course centered around application of IMFs and culminating in an individual lab practical as an authentic assessment provides several key benefits: straightforward articulation of learning outcomes and evidence of student learning through the practical itself; a clear target that all activities can be intentionally scaffolded toward; promotion of greater depth of understanding versus broad topical coverage; and alignment of course goals with student motivations which fosters eagerness to master the necessary skills. We propose that other institutions may find value in adopting a similar backward design approach, with a central theme, when seeking to create more engaging laboratory courses. While the specific approaches that work in our context may not be effective at all institutions, many alternative approaches could serve the same function. Our iterative, collaborative course improvement process produced meaningful growth for both students and faculty members.

## Supporting Information Available

Student Handout Lab Practical (PDF)
Orchestrated Slides for Lab Practical (PDF)
Instructor-Facing Process Checklist (PDF)
Student-Facing Holistic Rubric for Grading the Lab Practical (PDF)
Example of student flow scheme concepts rated NOT Well Demonstrated (PDF)
Example of student flow scheme concepts rated Well Demonstrated (PDF)
Interview transcripts and ratings for two example students (PDF)
Orchestrated Slides for Solubility of Chemotherapy Drugs Lab (PDF)
Instructor Notes and Timeline for the Solubility of Chemotherapy Drugs Lab (PDF)
