# Supplementary material for: Cultivating a Meaningful Application of IMFs through Backward Laboratory Course Design
Source: J Chem Educ. 2024 May 8;101(6):2331–42. doi: 10.1021/acs.jchemed.3c00810 (PMC11171254; doi:10.1021/acs.jchemed.3c00810)
Supplement: Supplementary file 1 — ed3c00810_si_001.pdf [file ed3c00810_si_001.pdf]

Cultivating a Meaningful Application of IMFs Through Backward Laboratory Course Design

Brenda B. Harmon[a]*, Deepika Das[a], Annette W. Neuman[a], Simbarashe Nkomo[a], Nichole L. Powell[a], Austin Scharf[a]

[a] Department of Chemistry, Oxford College of Emory University, Oxford, GA 30054, United States
*Email: bharmon@emory.edu

Separation and Recovery of Caffeine from a Commercial Product C202L

During the lab practical you will work independently to separate and recover the caffeine from a commercial product (a mixture of substances in either tablet, powder, or aqueous solution form). During the lab practical you will analyze your recovered caffeine to confirm identity and test purity. The lab practical is meant to encourage personal responsibility in the laboratory and to give you some individual feedback on fundamental laboratory techniques and thinking skills that you will need in future laboratory situations (chemistry, biology, biochemistry, neuroscience, pharmacology, etc).

Do not be nervous about the lab practical…since we have been repeating and reinforcing all of the techniques involved, we expect that every student who has been working to master the concepts and techniques will do well. You do not need to be “perfect” to do well on the lab practical. This is your opportunity to be challenged by working in the laboratory without a partner and use all of the techniques and thinking skills you have learned this semester.

The lab practical grade will be based on a qualitative evaluation of your process skills, the choices you make during the procedure that indicate conceptual understanding, the independence you show in being able to perform and think through the procedure, and your ability to demonstrate good lab practices. A smaller portion of the evaluation will include a quantitative evaluation based on the effectiveness of the separation (the amount of caffeine recovered from the pill).

Safety: Safety glasses should be worn at all times while working in the laboratory. This experiment may involve a strong base (1 M NaOH) or a strong acid (1M HCl) which are corrosive to eyes and skin. The organic solvents available for use in this experiment are all flammable. Wear gloves when handling all chemicals in this experiment. The TLC mobile phase contains dichloromethane which is a suspect carcinogen. Use only in the hood and do not get on your skin.

Waste: Aqueous waste solutions should go in the aqueous waste container and organic waste solutions should go in the organic waste container. If the waste containers in your hood are full, tell a TA or the instructor.

I. Purpose/Goal of the lab:

To separate and recover caffeine from a consumer product with high recovery and high purity.

Once you have completed separation and recovery, you will be left with two Big Questions:

- ***How does the amount of caffeine you recovered compare to the amount of caffeine reported in the product by the manufacturer?***
- ***Is the white solid you recovered caffeine and is it pure?***

***YOU WILL RECORD your abbreviated PROCEDURE (include only the important details) and ALL DATA, OBSERVATIONS, and CONCLUSIONS ON THE FLOW SCHEME.*

You will be given a large piece of paper to use for your flow scheme/lab notebook with the structures of the ingredients in your assigned consumer product drawn for you at the top.

Abbreviated OUTLINE of a General Procedure

- **If your consumer product is a solid**: Add the contents of the vial containing your sample to a 50-ml beaker. Add 15 ml of an aqueous phase of your choice (1M NaOH (aq), 1 M HCl (aq), 10% NaCl(aq)-*brine* , and DI water will be available to you). Use some of your chosen aqueous phase to rinse the inside of the vial to make sure you get all of the sample into the solution. Record your observations. Continue with the procedure starting with step 1.

- **If your consumer product is a liquid**: Add the contents of the vial containing your sample to a 50-ml beaker. Add 10 ml of an aqueous phase of your choice (1M NaOH (aq), 1 M HCl (aq), 10% NaCl(aq)-*brine*, and DI water will be available to you). Use some of your chosen aqueous phase to rinse the inside of the vial. Record your observations. Continue with the procedure starting with step 1.

1) Carefully pour your consumer product solution into a separatory funnel, and extract using a total of 30 ml of an organic solvent of your choice (you will be

provided with ~35 ml of five different organic solvents in your hood). Keep in mind that your goal is to recover all of the caffeine in the powder at the end of this procedure. Once finished, combine all of the organic layers into the same labeled container.

2) Pour the organic phase back into the separatory funnel. Wash using 10 mL of a chosen aqueous phase. Repeat if necessary.

3) Dry the organic layer over your choice of anhydrous drying agent. *Show the instructor before moving to the next step.

4) Transfer the "dry" organic solvent by decanting, making sure to leave the solid drying agent behind, into a small round-bottom flask (rbf). Rinse the solid drying agent with two small portions of the same organic solvent, in order to make sure that you get every molecule of caffeine out of the beaker and into the rbf.

5) Perform TLC analysis of the organic solution. Use silica gel coated plates, each student will be provided with the appropriate mobile phase based on the molecules in their consumer product. Show your instructor your TLC plate under the lamp. (Record the TLC experiment on the flow scheme)

1) Why did you run the TLC plate? What is your QUESTION?
2) What were the stationary and mobile phases? (so you or someone else could repeat the work later)
3) What did the TLC plate tell you? (conclusion)*

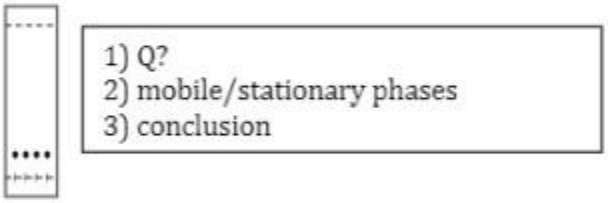

6) Evaporate the organic solvent using rotary evaporation. **Your instructor will supervise your use of the roto-vap and help you (if necessary)*. Hopefully, this will yield caffeine as a white solid.

7) Run a melting point experiment to test the purity of your (hopefully) white solid. **Your instructor will help you set up the device (if necessary)*. Record the mp experiment on your flow scheme/lab notebook sheet.

DO NOT THROW AWAY YOUR CAFFEINE!

Use the bent thin metal spatula to scrape out as much of the solid as you can. Put the vial in the sealed bag and write your name and lab section on the bag using a sharpie before you give it to your instructor for grading.

Finally - Summarize the important quantitative data in a simple, brief table and answer the beginning questions in your notebook.

How does the amount of caffeine you recovered compare to the amount of caffeine reported in the powder?

HINT: this comparison is always best represented by contextualizing amounts as % (for example: % error in measurement, % yield in synthesis, and % loss or % recovery for separations).

Is the white solid you recovered caffeine and is it pure?

**Turn in the Flow Scheme/lab notebook sheet and your recovered solid as you leave the lab.

Cleanup: All organic waste should go in the organic waste container. All aqueous waste should go in the acid/base waste container.
