# Supplementary material for: Cultivating a Meaningful Application of IMFs through Backward Laboratory Course Design
Source: J Chem Educ. 2024 May 8;101(6):2331–42. doi: 10.1021/acs.jchemed.3c00810 (PMC11171254; doi:10.1021/acs.jchemed.3c00810)
Supplement: Supplementary file 2 — ed3c00810_si_002.pdf [file ed3c00810_si_002.pdf]

CULTIVATING A MEANINGFUL APPLICATION OF IMFS THROUGH BACKWARD LABORATORY COURSE DESIGN

- BRENDA B. HARMON[A]*, DEEPIKA DAS[A], ANNETTE W. NEUMAN[A], SIMBARASHE NKOMO[A], NICHOLE L. POWELL[A], AUSTIN SCHARF[A]
- [A] DEPARTMENT OF CHEMISTRY, OXFORD COLLEGE OF EMORY UNIVERSITY, OXFORD, GA 30054, UNITED STATES
- *EMAIL: BHARMON@EMORY.EDU

[redacted]’S HEADACHE RELIEF SHOTS

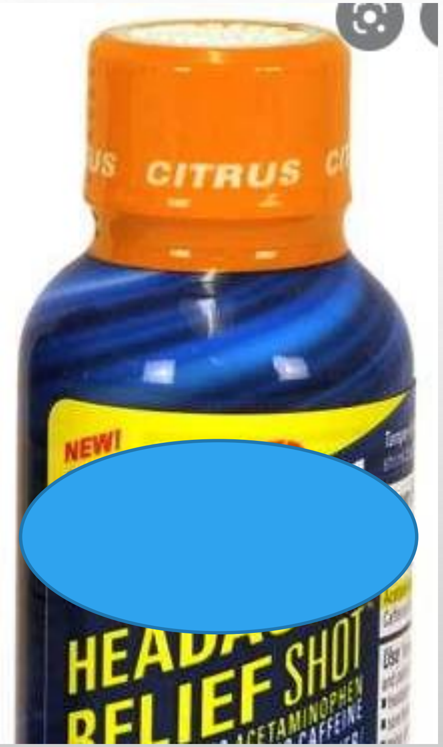

CHEM 202L LAB PRACTICAL

A CULMINATING EXPERIENCE FROM THE BEGINNING OF THE SEMESTER, TO DEMONSTRATE WHAT YOU HAVE LEARNED (COMBINING TECHNIQUES, CONCEPTS, AND SCIENTIFIC THINKING SKILLS) AND TO HELP YOU LEARN EVEN MORE. •

THE HONOR CODE APPLIES TO THIS QUALITY CHECK. **DO NOT TALK TO ANOTHER STUDENT DURING THE LAB**.

QUESTIONS FOR THE INSTRUCTOR ARE ENCOURAGED AND ARE IMPORTANT. BE AWARE THAT YOUR ABILITY TO DEMONSTRATE YOUR INDEPENDENCE AND TIME MANAGEMENT IS PART OF YOUR GRADE.

Goody's Headache Relief Shots FLOW SCHEME

Thinking and writing in terms of structures is an indication that you have developed a more robust and sophisticated understanding of the Chem 202 course content. ***Remember, your macroscale procedure is only possible due to what is happening at the molecular level.***

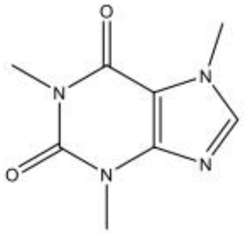

caffeine

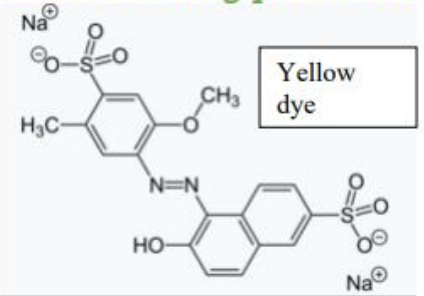

Yellow dye

One serving:

- Caffeine = 50 mg per dose
- ***You have been given an amount of the liquid equal to one dose.**

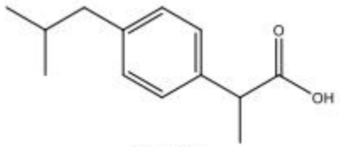

ibuprofen

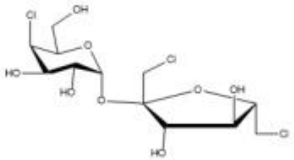

sucralose

**You only need to re-draw the structures if the molecule undergoes a CHEMICAL change. Otherwise, you can use the name.*

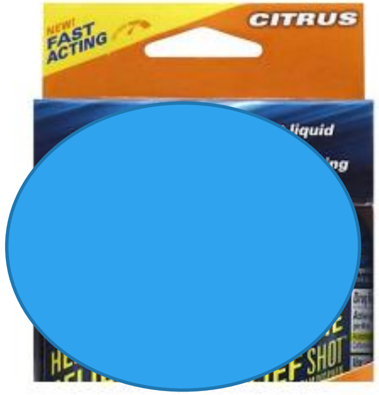

DRAW A SIMPLE FLOW SCHEME/PROCEDURE

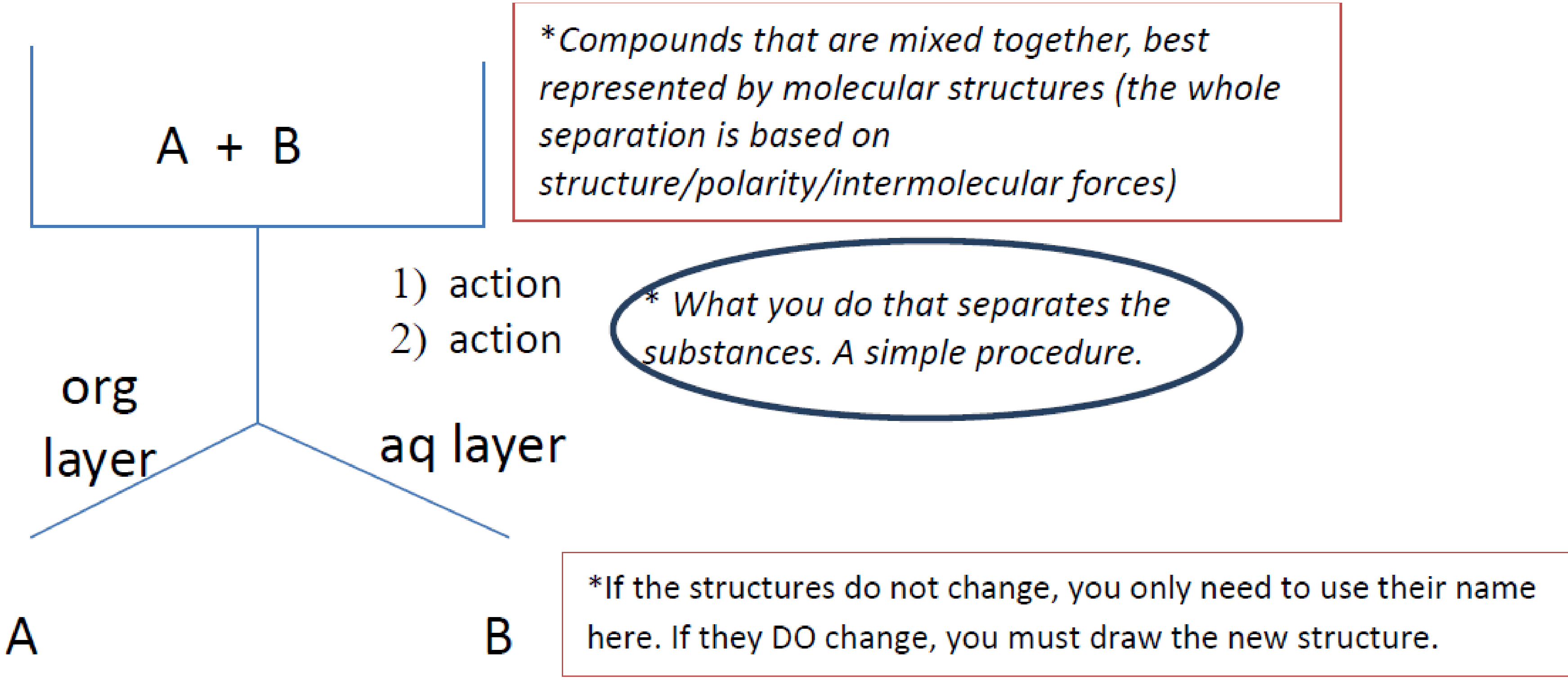

Figure 2. Outline of a Simple Flow Scheme

**Compounds that are mixed together, best represented by molecular structures (the whole separation is based on structure/polarity/intermolecular forces)*

** What you do that separates the substances. A simple procedure.*

*If the structures do not change, you only need to use their name here. If they DO change, you must draw the new structure.

TLC ANALYSIS

MOBILE PHASE IS ETHYL ACETATE

STANDARDS AVAILABLE TODAY:

- C= CAFFEINE
- I = IBUPROFEN
- S = SUCRALOSE

CLEAN UP

- EMPTY AQUEOUS WASTE INTO PROPER CONTAINER
- DISSOLVE DRYING AGENT IN SMALL AMOUNT OF WATER AND PUT INTO AQUEOUS WASTE
- PUT USED CAPILLARY SPOTTERS IN THE GLASS DISPOSAL BOX.
