# Supplementary material for: Cultivating a Meaningful Application of IMFs through Backward Laboratory Course Design
Source: J Chem Educ. 2024 May 8;101(6):2331–42. doi: 10.1021/acs.jchemed.3c00810 (PMC11171254; doi:10.1021/acs.jchemed.3c00810)
Supplement: Supplementary file 3 — ed3c00810_si_003.pdf [file ed3c00810_si_003.pdf]

Cultivating a Meaningful Application of IMFs Through Backward Laboratory Course Design

Brenda B. Harmon[a]*, Deepika Das[a], Annette W. Neuman[a], Simbarashe Nkomo[a], Nichole L. Powell[a], Austin Scharf[a]

[a] Department of Chemistry, Oxford College of Emory University, Oxford, GA 30054, United States
*Email: bharmon@emory.edu

Instructor-Facing Process Checklist

Hood	Name	Safety *appropriate clothes *shoes *hair tied back if needed *safety glasses	Sep Funnel Use *lid is stabilized when inverting) *burping *shaking	Drying Agent Use * not way too much * no water droplets visible *can explain what they were looking for	TLC *design *execution (correct mobile phase, spotting line is not below mobile phase, choice of standards) *analysis	Melting Point *assessed via NB entries (literature value in NB, more than one sample, recorded as a range, analysis)	Independence * types of questions students asked during the practical	Time
1								
1								
2								
2								

To mitigate the difficulties inherent in observing and evaluating multiple students performing the same experiment at the same time, no more than 12 students were evaluated during a lab practical session and each instructor was supported by a dependable TA who used the process checklist to provide a second assessment of safety, sep funnel use, independence, and time. The instructor and TA conferred regularly during the session to make sure they were in agreement with observations. The students were required to interact with the instructor after using the drying agent and after performing TLC. During this interaction, the instructor was able to observe the results of the student process and ask questions to probe understanding.

In the determination of a **PROCESS** rating (the ability to perform the laboratory procedure):

Instructors in the course negotiated norms and process list criteria throughout the first two implementations of the lab practical. Faculty agreed that we were NOT expecting expert technique as our students are only first- or second-year undergraduate students.

To meet the competency threshold, students needed to isolate enough caffeine to take a TLC and do this independently (with little or no direct support for: making choices, using the sep funnel, identifying which layers are which, using the drying agent, and taking a TLC). Students could ask any questions they felt they needed to ask, but they knew that independence was being evaluated. Any unusual issues, such as an emulsion, or other types of questions (like clarification questions) did not factor against demonstrated independence.

Major criteria (norms):

- Sep Funnel - lid must be stabilized when upside down; shaking must occur (not just burping)
- Drying agent – no visible signs of water; not too large an amount of drying agent (covering the entire bottom of the beaker)
- TLC – if two or more criteria was missing or at issue, this was considered to be a major issue
- Mp- must be recorded as a range

Minor criteria (norms):

- Explanation of how to know when you had added enough drying agent was considered weak
- Asked for a second round-bottom flask, as they forgot to take the mass
- Only one of the TLC criteria was missing or at issue
- Asked for a third TLC plate
- Melting point – only one sample; literature value not recorded

*Safety, Independence, and Time were factored in after the process rating was determined. These factors could influence the overall grade.

We provided students with feedback on a different, more holistic rubric using what was recorded on this process checklist (available in Supplemental Materials)

- A rating of **Well Demonstrated** indicated that a student met the process checklist criteria with only 2-3 minor issues or omissions -**or**- one major issue or omission across the categories.
- A rating of **Well Demonstrated With a Few Issues** indicated that a student met the criteria with two major issues or omissions and possibly another minor issue across the process categories.
- A rating of **Not Well Demonstrated** indicated that there were at least two major issues and possibly several minor issues demonstrated across the process categories.

Students rated as **Not Well Demonstrated** often demonstrated more than one of the following behaviors:

- started the process much later than their peers (re-read the instructions over and over before starting lab work)
- had difficulty handling the separatory funnel; held it completely upside down without stabilizing the lid
- had difficulty identifying which layer was which (tried to dry and evaporate the aqueous layer)
- made multiple errors in running a TLC plate (the mobile phase was higher than the line of origin, used an mobile phase that did not separate the substances, did not spot reference standards, spotted all the reference standards instead of just those that were in their given mixture)
