# Supplementary material for: Cultivating a Meaningful Application of IMFs through Backward Laboratory Course Design
Source: J Chem Educ. 2024 May 8;101(6):2331–42. doi: 10.1021/acs.jchemed.3c00810 (PMC11171254; doi:10.1021/acs.jchemed.3c00810)
Supplement: Supplementary file 4 — ed3c00810_si_004.pdf [file ed3c00810_si_004.pdf]

Cultivating a Meaningful Application of IMFs Through Backward Laboratory Course Design

Brenda B. Harmon[a]*, Deepika Das[a], Annette W. Neuman[a], Simbarashe Nkomo[a], Nichole L. Powell[a], Austin Scharf[a]

[a] Department of Chemistry, Oxford College of Emory University, Oxford, GA 30054, United States
*Email: bharmon@emory.edu

Chemistry 202L Lab Practical Rubric

The isolation of caffeine from a consumer product is a culmination of multiple weeks of laboratory experiences encompassing important techniques and course concepts:

- IMFs
- Solubility
- Liquid–liquid extraction
- Equilibrium
- Using sequential extractions to manipulate the partitioning equilibrium
- Using acid–base chemistry to change structure, polarity, and solubility preferences in a molecule
- Practicing a molecular perspective to identify and explain what happens in the acid–base reaction
- Designing and executing a TLC experiment to analyze your results

Your individual performance on this experiment was evaluated holistically by your ability to complete the process of the laboratory procedure as well as how well you demonstrated your understanding of the concepts underlying the procedure.						
	**A**	**A-/B+**		**B/B+**	**B**	**C**
**Process**	✓	✓-	✓	✓-	✓	✓--
**Conceptual**	✓	✓	✓-	✓-	✓--	✓--
**Overall evaluation**	**95**	**88–92**		**85–87**	**83–85**	**75**

Legend: ✓ Well demonstrated ✓- Demonstrated with a few issues ✓-- Not well demonstrated

Process: Your demonstrated ability to see and perform the laboratory procedure at the *observable* level (what your eyes can see and your hands do)
Conceptual: Your demonstrated understanding of how course concepts apply to the procedure at the *molecular* level (where your eyes can't see)

The macroscale procedure is only possible due to what's happening at the molecular level. Effective connecting the two is what practicing laboratory science is all about!

Other factors that influence your score:

**Independence** *(expected)*<br>*up to -10 pts*	
**Time management** *(expected)*<br>*up to – 5 pts*	
**Safety** *(expected)*<br>*p to -10 pts*	
**Visual Quantity Recovered**<br>avg(expected)<br>below average (up to -5 pts)	

Total score

Process: Your demonstrated ability to see and perform the laboratory procedure at the *observable* level (what your eyes can see and your hands do)

**Overall process evaluation**	You seemed well-prepared and demonstrated a clear understanding of all aspects of the project. Good job!
	You seemed prepared and demonstrated a clear understanding of most aspects of the process.
	You did not seem well-prepared for performing the techniques and procedure independently

Areas for improvement: Did you…

- Handle the separatory funnel in a way that showed you understood what was important?
- Use the drying agent in a way that showed you understood how it worked?
- Correctly design, execute, and analyze your TLC experiment?
- Independently apply the procedural details and obtain enough solid product for TLC and melting point?
- Report your procedure using the flow scheme convention with enough detail that someone else could repeat your lab work?
- Extract multiple times with the correct phase?
- Wash with the correct phase?
- Obtain a yield under 125%?
- Manage your time effectively?
- Use good laboratory practices?

Conceptual: Your demonstrated understanding of how course concepts you have studied all semester apply to the procedure at the *molecular* level (where your eyes can't see)

**Overall conceptual evaluation**	You demonstrated the ability to pull together big course concepts and ideas. Good job!
	You demonstrated the ability to pull together some of the big course concepts and ideas.
	You didn't seem to understand what was happing on the molecular level (where your eyes can't see).

Areas for improvement: Did you…

- Choose an appropriate organic phase and explain why you chose it?
- Choose an appropriate aqueous phase and explain why you chose it?
- Clearly explain the molecular consequences of your aqueous phase choice?
- Predict where all components of the mixture end up and explain why?
- Explain how your procedural choices maximized the amount of substance you isolated?
- Use your data to accurately assess the quantity and purity of your isolated substance?
