# Supplementary material for: Cultivating a Meaningful Application of IMFs through Backward Laboratory Course Design
Source: J Chem Educ. 2024 May 8;101(6):2331–42. doi: 10.1021/acs.jchemed.3c00810 (PMC11171254; doi:10.1021/acs.jchemed.3c00810)
Supplement: Supplementary file 5 — ed3c00810_si_005.pdf [file ed3c00810_si_005.pdf]

# Cultivating a Meaningful Application of IMFs Through Backward Laboratory Course Design

Brenda B. Harmon[a]*, Deepika Das[a], Annette W. Neuman[a], Simbarashe Nkomo[a], Nichole L. Powell[a], Austin Scharf[a]

[a] Department of Chemistry, Oxford College of Emory University, Oxford, GA 30054, United States
*Email: bharmon@emory.edu

**Tylenol Headache Powder** FLOW SCHEME

*Thinking and writing in terms of structures is an indication that you have developed a more robust and sophisticated understanding of the Chem 202 course content.* ***Remember, your macroscale procedure is only possible due to what is happening at the molecular level.***

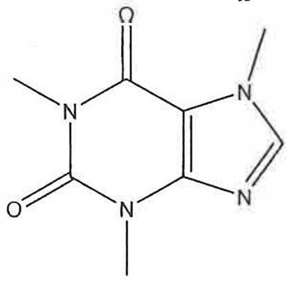

caffeine

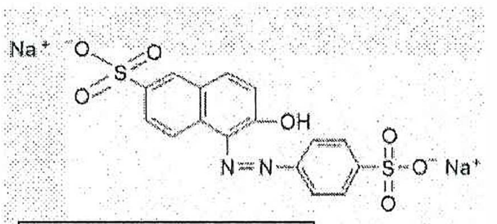

FD&C red dye #40

Active ingredients per dose: 60mg × 1g/1000mg = 0.06
- caffeine 60 mg
- ibuprofen 50 mg

***You have been given a single dose.**

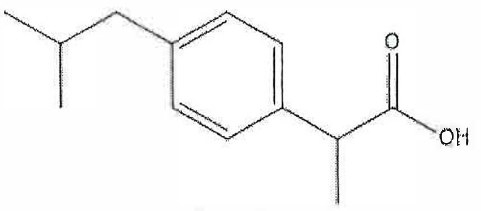

ibuprofen

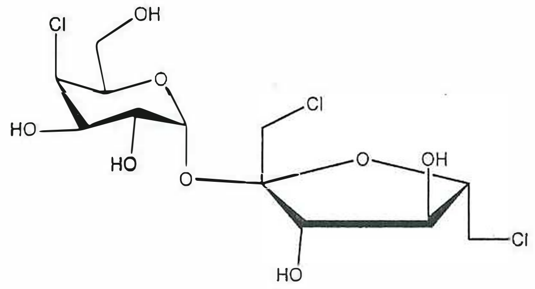

sucralose

## Procedure:

1.) Add solid drug sample to 50mL beaker w/ 15mL DI water

2.) Add Aqueous solution to the sep funnel then add 10mL of DCM to the sep funnel & perform a liq-liq extraction draining the organic phase 3 times, so you use a total of 30mL of DCM for the extraction

3.) Once the organic has been extracted & drained 3 times, drain aq phase in a separate beaker then wash organic solution in sep funnel w/ 10mL NaCl brine & drain organic & brine separately

4.) Decant "dry" organic solvent into rbf

5.) Peform a TLC analysis w/ 2 spots of the organic solution & one spot of the caffeine standards solution

6.) Evaporate the organic solvent using roto-vap then measure mass of rbf w/ solid caffeine

7.) Run a melting point experiment of white solid to test its caffeine purity

## Observations:

* aq phase → DI water

* 0.06g of caffeine per dose

- when water was added to sample, solution turned red

* organic solvent → DCM

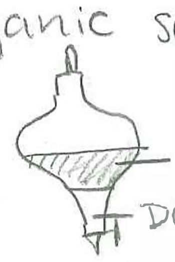


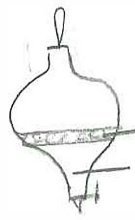


* wash solution w/ NaCl brine

* drying agent → $MgSO_4$

mass of rbf empty: 30.035g

C = caffeine standards

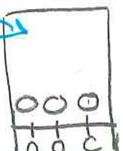


Q: Does the organic solution contain caffeine?

Mobile: Ethyl Acetate

Stationary: Silica gel

C: Organic Solution contains caffeine.

* Accepted Melting Point of Caffeine = 228°C

* Accepted Melting Point of caffeine as a hydrate = 234°C

Mass of rbf + caffeine: 30.115g

* after evaporating DCM caffeine is a white solid in the rbf

* Melting Point (experiment) 223-225°C

- solid is pure

Mass of caffeine

30.115g - 30.035g = 0.08g caffeine

* organic layer

DCM + caffeine

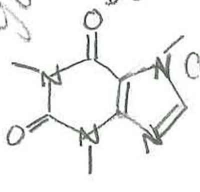

* aqueous layer

DI water +

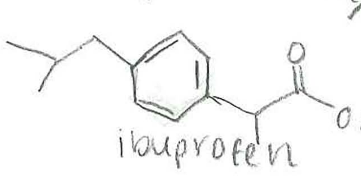

ibuprofen

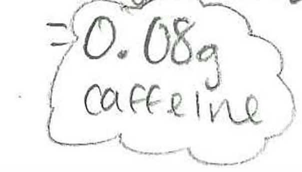

## Conclusions & Data:

The original sample of the drug contained 0.06g of caffeine and after extracting caffeine w/ liq. liq. extraction & evaporating it, I recovered 0.08g of caffeine.

$$\% \text{ error} = \left|\frac{\text{actual} - \text{theoretical}}{\text{theoretical}}\right| \times 100$$

$$= \left|\frac{0.06 - 0.08}{0.08}\right| \times 100$$

$$= 25\% \text{ error}$$

Since my % error is relatively low, the substance I obtained is mostly pure caffeine but contains an extra amount of outside sources due to the mass being higher than the original. The higher mass could also be due to the sensitivity of the balance.

The organic solvent I chose proved to extract caffeine from the aqueous solution (water) and resulted in a white solid substance that also proved to be caffeine b/c of its melting point.
