# Supplementary material for: Cultivating a Meaningful Application of IMFs through Backward Laboratory Course Design
Source: J Chem Educ. 2024 May 8;101(6):2331–42. doi: 10.1021/acs.jchemed.3c00810 (PMC11171254; doi:10.1021/acs.jchemed.3c00810)
Supplement: Supplementary file 6 — ed3c00810_si_006.pdf [file ed3c00810_si_006.pdf]

Cultivating a Meaningful Application of IMFs Through Backward Laboratory Course Design

Brenda B. Harmon[a]*, Deepika Das[a], Annette W. Neuman[a], Simbarashe Nkomo[a], Nichole L. Powell[a], Austin Scharf[a]

[a] Department of Chemistry, Oxford College of Emory University, Oxford, GA 30054, United States
*Email: bharmon@emory.edu

Children's Tylenol Dissolve Pack FLOW SCHEME

Thinking and writing in terms of structures is an indication that you have developed a more robust and sophisticated understanding of the Chem 202 course content. ***Remember, your macroscale procedure is only possible due to what is happening at the molecular level.***

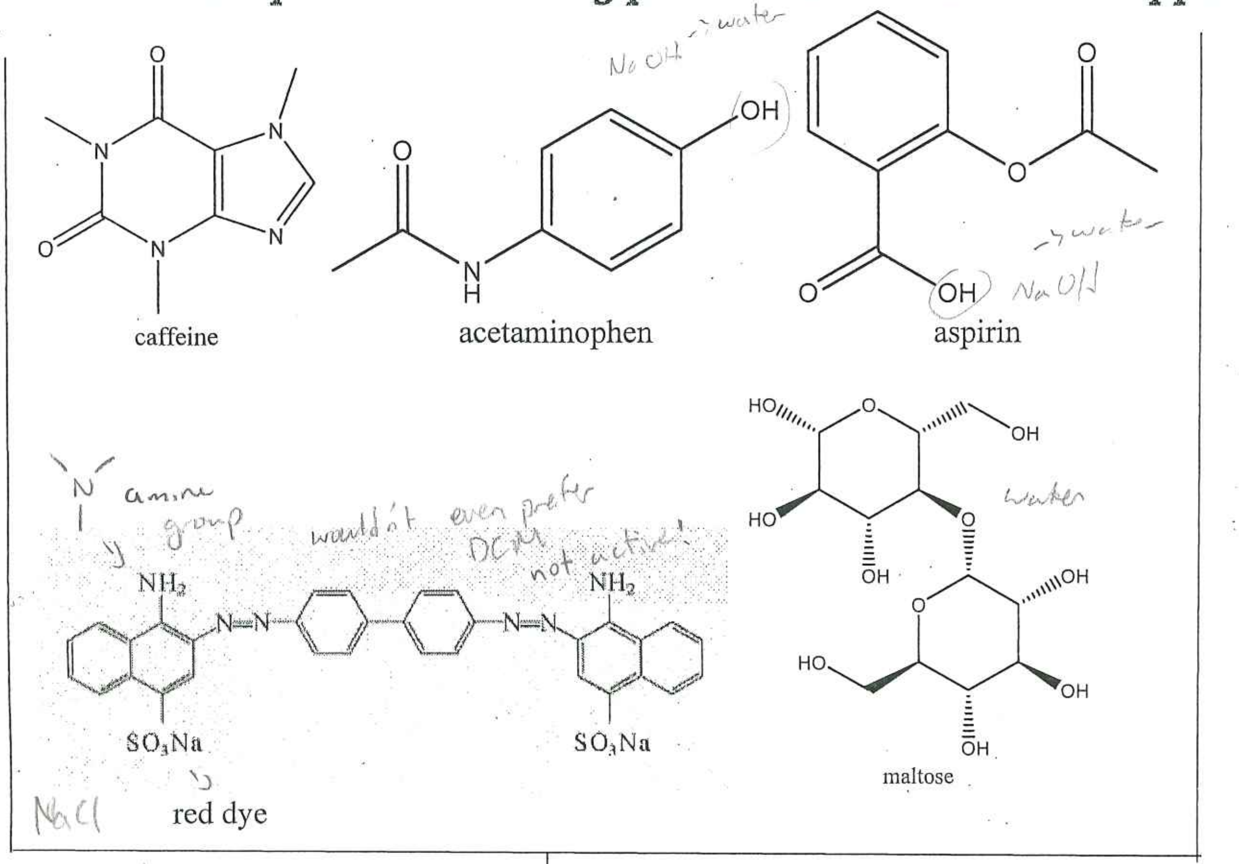

Active ingredients in a single dose packet:
- acetaminophen 60 mg
- aspirin 60 mg
- caffeine 60 mg

***You have been given a single dose sample**

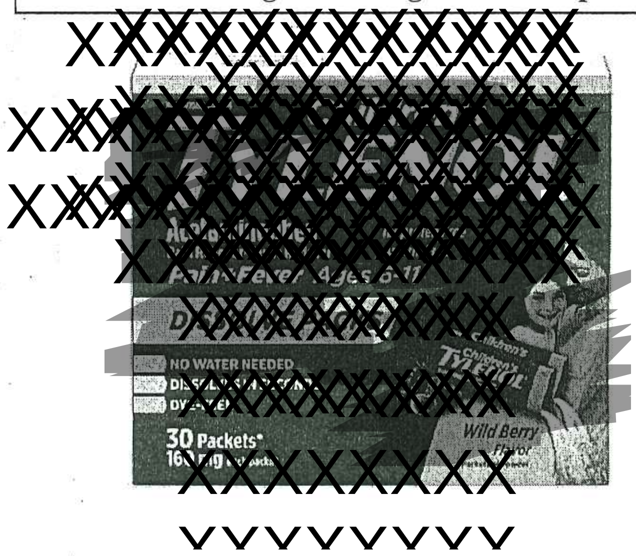

~~Active ingredients~~

acetaminophen, Na^{+}

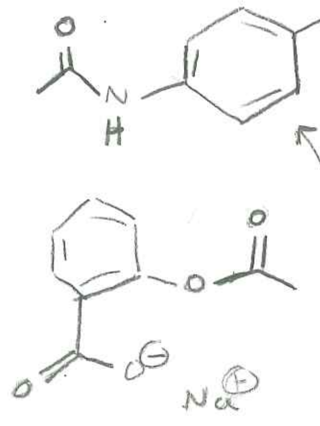

aspirin,

maltose, red dye (no structure changes)

aqueous phase

aq phase residual other ingredients

Use 15 mL NaOH to dissolve Tylenol in a 50 mL beaker

Use 10 mL DCM to perform liquid-liquid extraction with NaOH solution (x3) (30mL total)

~~29.203~~
~~29.244~~

↓

organic phase; caffeine (same structure)

wash organic phase with 10 mL NaCl solution (liq-liq extraction)

↓

organic phase: caffeine

Dry organic phase with $MgSO_4$ anhydrous

↓

organic phase: caffeine + DCM

Perform TLC on organic phase →

use rotovap to extract caffeine from organic phase (DCM)

Take melting point of leftover solute to test for purity ↳ (Melting point: 233° — 235°C) 2° melting range, pure

Plateau temp: 224°C

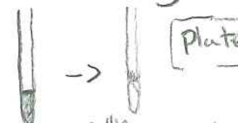

TLC Plate

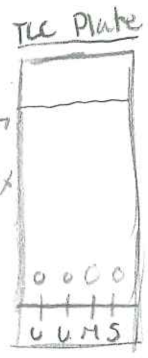

U= unknown/tested substance
M= co-spotted. Mix of standard and unknown
S= standard

Question: Is the caffeine pure?

Phases: stationary: Silica gel
Mobile: Ethyl Acetate

Conclusion ~~Inference~~: The caffeine is pure, as the standards spot, the co-spot, and both ~~test~~ unknown spots all had the same Rf value, and therefore the same level of attraction between phases.

Rotovap Results:

~~Mass~~	mass(g)
Regular RBF	29.203
RBF with solute	29.244
Solute	0.041

organic phase / leftover solute

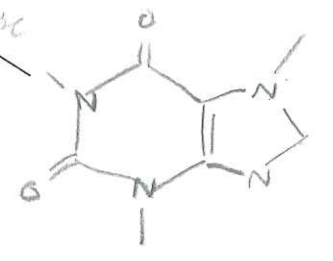

Answer to beginning Question:

I was able to recover around 68% of the total 60 mg of caffeine in the product. This is not an exceptionally high yield, though this does mean that 41 mg were recovered. The caffeine seemed to be pure through use of TLC and melting point. TLC showed that the standards spot and the tested spot had the same rf value. The melting range was also 2°C, which is within the range to melt sharply, demonstrating that the substance recovered was likely pure caffeine.

Results:

~~solute (caffeine)~~	melting point (°C)	Mass (mg)	% yield
caffeine	233-235	~~0~~41	$\frac{41}{60} \times 100 = 68\%$

Around 68% of the initial 60 mg of caffeine were recovered. The caffeine appeared to be slightly flaky.

Qualitative Recordings:

	color:	what was present
Aqueous phase:	Turned blue (indicator)	acetaminophen, aspirin, maltose, red dye
Organic phase:	Turned red (indicator)	caffeine

caffeine:

state:	Visual:
solid:	white flakes
liquid:	orange-like liquid
