# Supplementary material for: Cultivating a Meaningful Application of IMFs through Backward Laboratory Course Design
Source: J Chem Educ. 2024 May 8;101(6):2331–42. doi: 10.1021/acs.jchemed.3c00810 (PMC11171254; doi:10.1021/acs.jchemed.3c00810)
Supplement: Supplementary file 7 — ed3c00810_si_007.pdf [file ed3c00810_si_007.pdf]

Cultivating a Meaningful Application of IMFs Through Backward Laboratory Course Design

Brenda B. Harmon[a]*, Deepika Das[a], Annette W. Neuman[a], Simbarashe Nkomo[a], Nichole L. Powell[a], Austin Scharf[a]

[a] Department of Chemistry, Oxford College of Emory University, Oxford, GA 30054, United States
*Email: bharmon@emory.edu

5-minute oral reflective interviews for evaluating conceptual understanding

Rating: NOT Well Demonstrated

Q: What organic solvent did you choose and why?

> Student: *DCM. Honestly, 'cause the previous experiment we did that said it was best at extracting caffeine from water. And then I used water as my aqueous phase.*

Q: Will DCM dissolve any of the other compounds in your mixture?

> Student: *No.*

Q: Will any of the compounds prefer DCM?

> Student: *Prefer DCM? Well, caffeine.*

Q: Just caffeine. So, your aqueous phase was plain water?

> Student:*Yes*.

Q: So tell me where everything went and why? You told me where the caffeine went, where did all the other components of your mixture go and why?

> Student: *Um…ibuprofen is in the aqueous phase because it hydrogen bonds with water.*
>
> *And then sucralose was the same. H-bonding.*
>
> *And then when I washed with the NaCl brine, that got rid of the dye because of ion-dipole and that went into the aqueous phase, too.*

Q: What part of the aqueous phase?

> Student: *The Na^+ Cl^-. They both ion-dipole.*

The rating for this student can be seen in the highlighted portions of Table S1 below.

Table S1. Instructor facing rubric for conceptual understanding.

Exemplary	Competent	Emerging	Novice
*Solvent **choices** make sense for the given mixture.*	*Solvent **choices** make sense for the given mixture.*	*Solvent choices make sense for the given mixture.*	*One or more solvent **choices** do not make sense for the given mixture.*
Justifications for solvent choices include use of molecular structural cues without prompting.	*Justifications for solvent choices include use of molecular structural cues after prompting.*	*Justifications may include some structural features after prompting but are not clearly connected to choices.*	*Justification may not include any mention of structural features, even after prompting.*
Justification for solvent choices includes effective language for IMFs without prompting.	*Justification for solvent choices may include effective language for IMFs after prompting.*	*Justification for solvent choices may include language for IMFs after prompting but the language choices may not be effective.*	*Narrative may include random use of IMF jargon (polarity) with no clear connection to choices.*

Solubility predictions make sense for the molecular structures and extraction system.<br><br>*Explanations are clear that preferential solubility was changed by altering structure, properties, and predominant IMFs.*<br><br>*May demonstrate integration of conceptual frameworks (for example: IMFs and acid/base).*	*Most solubility predictions make sense for the molecular structures and extraction system.*<br><br>*Explanations of acid/base reactions are meaningful, but do not explicitly include the need to use acid/base reaction to change solubility.*<br><br>*May demonstrate disjointed conceptual frameworks*	*Some solubility predictions make sense for the molecular structures and extractions system.*<br><br>*May seem lost in trying to identify and explain the details of acid/base reactions. Explanations not clear that water is the solvent.*<br><br>*No clear demonstration of either conceptual framework.*	*Predictions of solubility do not make sense for the molecular structures.*<br><br>*Explanations do not mention acid/base.*<br>*May not be able to identify aqueous or organic layers.*<br><br>*No use of either conceptual framework*

This corresponds toTable 7 in the manuscript.

Rating: Well Demonstrated

Q: What organic solvent did you choose and why?

Student: *DCM. When we did liquid-liquid extraction in a previous experiment. The caffeine had an affinity for the DCM due to similar IMFs – dipole-dipole. Essentially water would push out the caffeine into the organic layer.*

Q: Why would water do that?

Student*: Because the water -water interactions, those are most thermodynamically favorable.*

Q: Alright, so. In this mixture, this packet, is there anything else that would also prefer to dissolve in DCM?

Student: *Ummm….I said perhaps ibuprofen because it has a lot of hydrocarbon. However, that is the reason I added NaOH. Because there is a carboxyl group in ibuprofen and if you deprotonate that hydrogen, you turn this ionic and then you get the ibuprofen into the aqueous layer.*

Q: If it becomes ionic, how does that give it an affinity for the aqueous phase?

Student: *Because water forms ion-dipole IMFs with the ion.*

Q: Where did this dye go and why?

Student: *It went into the NaOH phase because* Na^{+} *dissociates and the* OH^{-} *forms ion-dipole forces with it. So, there's water in NaOH, and so that FDC red dye is ionic and the* O^{-2} *which will have a strong affinity for it. Making ion-dipole with the aqueous phase.*

Q: So what part of the aqueous phase does the FD&C red dye interact with?

Student*: Ummm (thinking for a few seconds)……it will have a strong affinity for the water. Ion-dipole forces with the water.*

Q: Alright, alright. Where did the sucralose go and why?

Student: *The sucralose went into the aqueous phase because sucrose forms very strong hydrogen bonding, so sucralose has a high affinity for water.*

The rating for this student can be seen in the highlighted portions of Table S2 below.

Table S2. Instructor facing rubric for conceptual understanding.

Exemplary	Competent	Emerging	Novice
*Solvent **choices** make sense for the given mixture.*	*Solvent **choices** make sense for the given mixture.*	*Solvent choices make sense for the given mixture.*	*One or more solvent **choices** do not make sense for the given mixture.*
Justifications for solvent choices include use of molecular structural cues without prompting.	*Justifications for solvent choices include use of molecular structural cues after prompting.*	*Justifications may include some structural features after prompting but are not clearly connected to choices.*	*Justification may not include any mention of structural features, even after prompting.*
Justification for solvent choices includes effective language for IMFs without prompting.	*Justification for solvent choices may include effective language for IMFs after prompting.*	*Justification for solvent choices may include language for IMFs after prompting but the language choices may not be effective.*	*Narrative may include random use of IMF jargon (polarity) with no clear connection to choices.*
Solubility predictions make sense for the molecular structures and extraction system.<br><br>*Explanations are clear that preferential solubility was changed by altering structure, properties, and predominant IMFs.*<br><br>*May demonstrate integration of conceptual frameworks (for example: IMFs and acid/base).*	*Most solubility predictions make sense for the molecular structures and extraction system.*<br><br>*Explanations of acid/base reactions are meaningful, but do not explicitly include the need to use acid/base reaction to change solubility.*<br><br>*May demonstrate disjointed conceptual frameworks*	*Some solubility predictions make sense for the molecular structures and extractions system.*<br><br>*May seem lost in trying to identify and explain the details of acid/base reactions. Explanations not clear that water is the solvent.*<br><br>*No clear demonstration of either conceptual framework.*	*Predictions of solubility do not make sense for the molecular structures.*<br><br>*Explanations do not mention acid/base.*<br>*May not be able to identify aqueous or organic layers.*<br><br>*No use of either conceptual framework*

This corresponds toTable 7 in the manuscript.
