# Supplementary material for: Cultivating a Meaningful Application of IMFs through Backward Laboratory Course Design
Source: J Chem Educ. 2024 May 8;101(6):2331–42. doi: 10.1021/acs.jchemed.3c00810 (PMC11171254; doi:10.1021/acs.jchemed.3c00810)
Supplement: Supplementary file 8 — ed3c00810_si_008.pdf [file ed3c00810_si_008.pdf]

Cultivating a Meaningful Application of IMFs Through Backward Laboratory Course Design

Brenda B. Harmon[a]*, Deepika Das[a], Annette W. Neuman[a], Simbarashe Nkomo[a], Nichole L. Powell[a], Austin Scharf[a]

[a] Department of Chemistry, Oxford College of Emory University, Oxford, GA 30054, United States
*Email: bharmon@emory.edu

Beginning Question: Predict the water solubility of a chemotherapy drug :

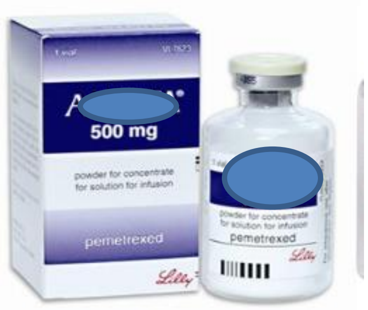

- ***High solubility in water***
- ***Low solubility in water***
- ***Mostly insoluble in water***
- ***Insoluble in water***

NOT simply:
Water soluble
Water insoluble

****NUANCED*

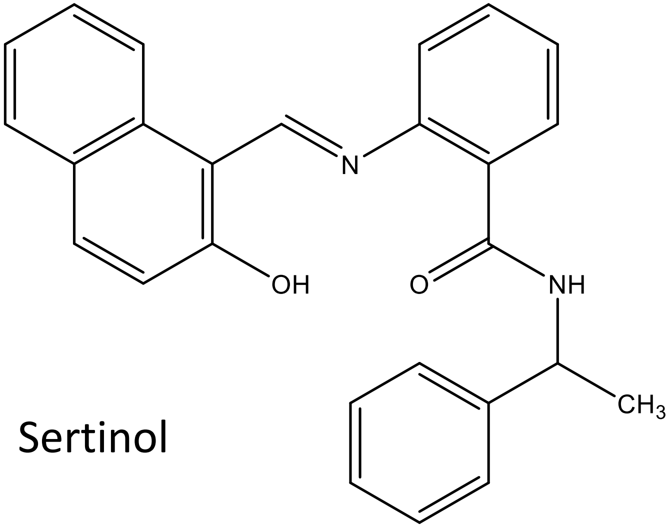

Sertinol

A relatively new chemotherapy drug that induces cell death in MCF-7 human breast cancer cells.

Why do you think the water solubility of a chemotherapy drug is important?

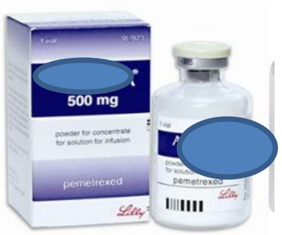

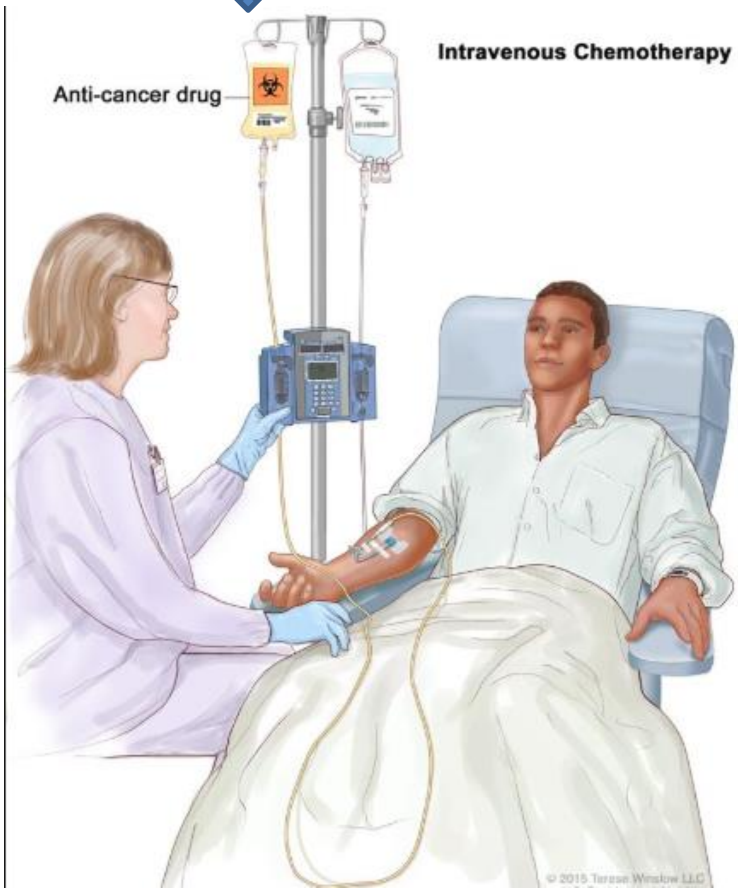

One of the most common ways to deliver chemotherapy drugs is through ***I****ntra****V****enous (IV) administration.*

Learning Goal for this lab session:

To be able to **predict the water solubility of organic molecules** by looking at their structures

- You will be individually assigned two chemotherapy drugs for your post-lab writing assignment.
- You will use what you learn from this lab session to help you predict the water solubility of each drug and rank them in order of water solubility.

T[illegible]l® (NSC 125973)

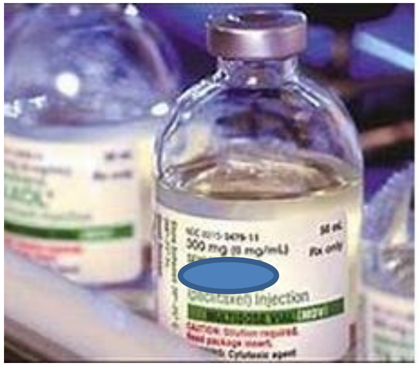

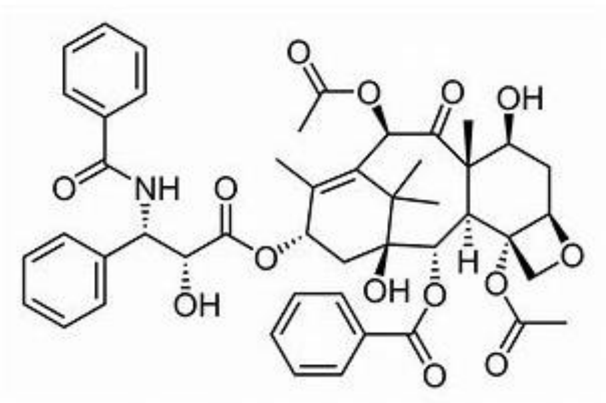

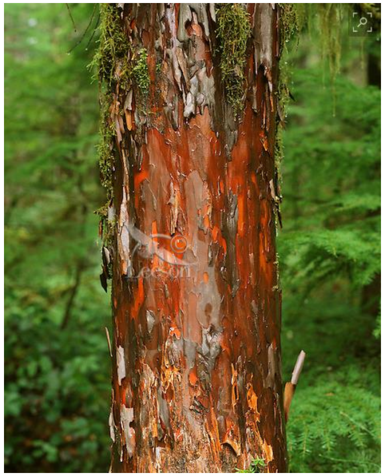

- The **most well-known and effective natural-source cancer drug** in the United States, is derived from the bark of the Pacific yew tree (*Taxus brevifolia*) and is highly potent in the treatment of breast, lung, and ovarian cancers.
- <u>**The biggest problem with Taxol is its low solubility in water.**</u> *Water solubility is important because a drug must be fully dissolved to be safe for injection, to be transported to the site of cancer cells, and to have a high enough dosage*. Any solid (undissolved) particles in an intravenous injection can lead to blood clots and inflammation of the veins. There are many efforts to increase Taxol's water solubility.

Source: National Cancer Institute https://dtp.cancer.gov/timeline/flash/success_stories/S2_taxol.htm

Overarching Goal
# of the three experiments today

- To identify relationships between
  - molecular structure and water solubility

Part A

Goal: Predict water solubility from structure

* *and identify mental models/thinking frameworks , see if they need to be changed or modified*

**why do we call these "organic" solvents?*

- dichloromethane (DCM)
- ethanol
- ethyl acetate
- hexane
- water

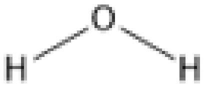

water

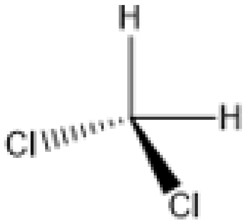

dichloromethane (DCM)

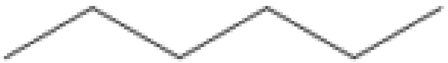

hexane

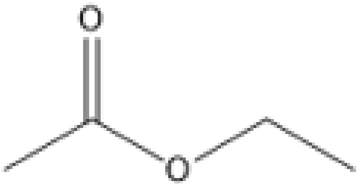

ethyl acetate

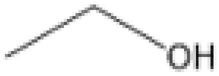

ethanol

Think/Pair/Share with your team

*Rank them in polarity - a *rough/relative ranking* (based only on structure)

Predicted Ranking

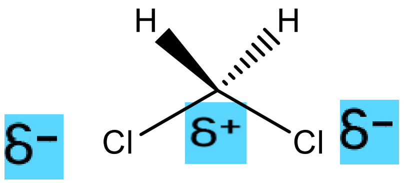

dichloromethane

? *We know it is polar, but where does it fit?*

**the experiment should clear-up any uncertainties*

most polar

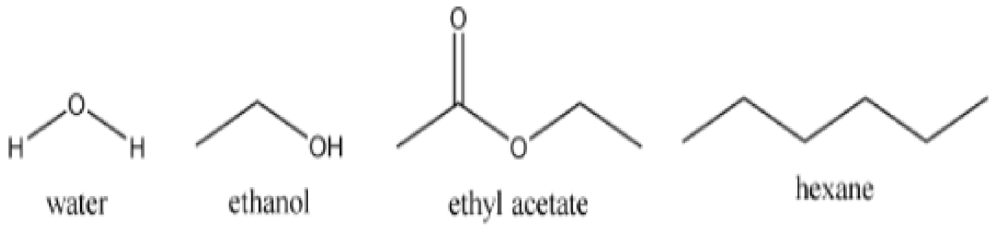

least polar

•Based on the structures and relative polarities, make an **individual prediction** as to which of the common organic solvents will be soluble in water.

•**RECORD YOUR PREDICTION on your handout.**

•**Your predictions may turn out to be wrong. That is okay! This is how science works and is a good way to learn.*

Discuss predictions with your group

- Report out -what are some of the theoretical frameworks or mental models that you used to make your water solubility prediction?

- *As we move through the lab session today, we will see if we need to modify or avoid certain mental models.*

We will ANSWER the question of water solubility through EXPERIMENTATION.

Part A: Procedural Details

1. Label a set of test tubes 1-5 using a sharpie and place each one into a small beaker.
2. Add water (approximately 1/3 of a test tube) to each test tube.
3. Add a different solvent to each test tube (approximately 1/3 of a test tube).
4. Use a pipet to mix the solvents and record all observations.

Experimental Overview

Investigate the miscibility of the organic solvents with water.
Assume that you will be provided with a set of small glass test tubes and with the solvents.

Identify variables:

- **Independent variable** (what varies?)
- **Dependent variable** (what is measured?)
- **Controlled variable(s)**

Pooled class data will provide replicates

Control experiment = ?

Experimental Overview

Investigate the miscibility of the organic solvents with water.
You will be provided with a set of small glass test tubes and with the solvents.
Work with your entire hood group.

Identify variables:

- **Independent variable** structure/polarity /IMFs of the organic solvent
- **Dependent variable** two layers/miscibility (practical solubility) in water
- **Controlled variable(s)** amounts, temp, etc

Pooled class data will provide replicates

Control experiment = water dissolved in water

Data for Part A

Table 1. Miscibility of some common solvents in water.

solvent 1	solvent 2	Observations •*no layers?* •*2 layers?* -*layer on top?* - *layer on bottom?* •*cloudy?*	Inference ('practical' solubility in water)
1water	water		
2water	ethanol		
3water	hexane		
4water	ethyl acetate		
5water	DCM		

**If the liquids form 2 layers, are the layers equal?*

*In this laboratory course, we care about 'practical' solubility or miscibility (qualitative).

Can you see two layers? Insoluble.
One layer? Soluble. **which layer is which?*

An ***observation*** is a descriptive statement about a natural phenomenon.

An ***inference*** is an interpretation of an observation.

RESULTS

**Creating meaning from the DATA*

Table 1. Water solubility and IMFs for 4 common laboratory solvents

Solubility	Solvent	IMFs with H_2O
Water soluble	ethanol 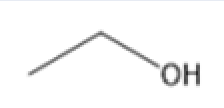	H-bonding<br>dip-dip<br>LDF
Water insoluble	hexane 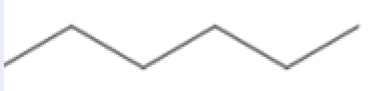<br>**top*	LDF
Water insoluble	DCM<br>*dichloromethane* 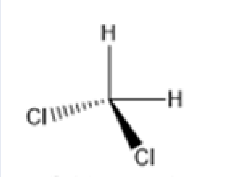<br>**bottom*	dip-dip<br>LDF

*****Were you surprised by any of the experimental results?**

From the results of the experiment: can you come up with a guideline to help predict water solubility based on structure?

Think/Pair/Share with the class

*For now, set ethyl acetate aside.

When predicting water solubility based on molecular structure:

A) If an organic molecule cannot H-bond with water, it will be immiscible with water.

LIKE IMFs DISSOLVE LIKE IMFs

*RECORD this discovery in your handout

??? *What's up with ethyl acetate?*

We'll answer that with more experimentation…

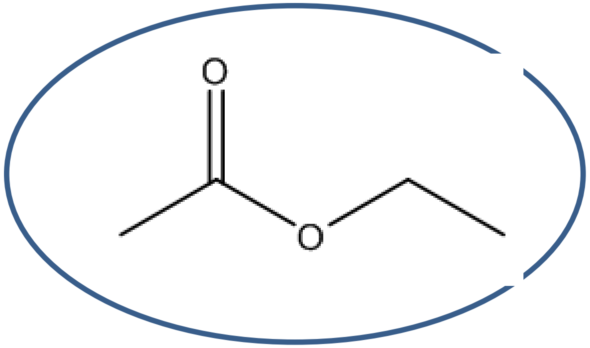

Rules for predicting H-bonds between two molecules

1) One of the molecules must contain a

H directly bonded to an O, N, or F atom

2) The other molecule must contain an

O, N, or F atom

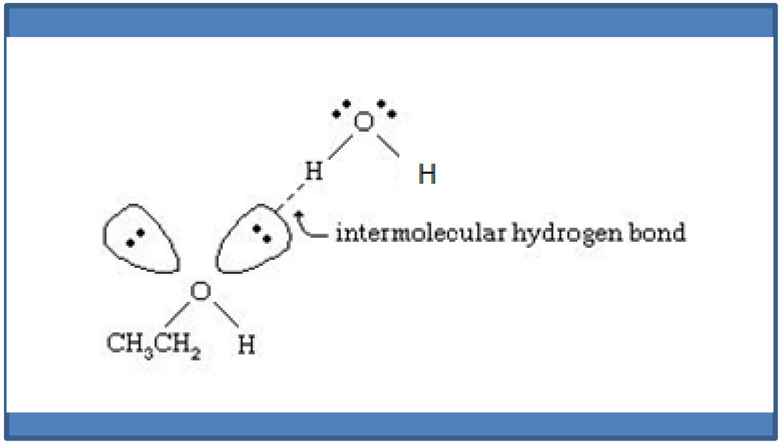

ethanol and water

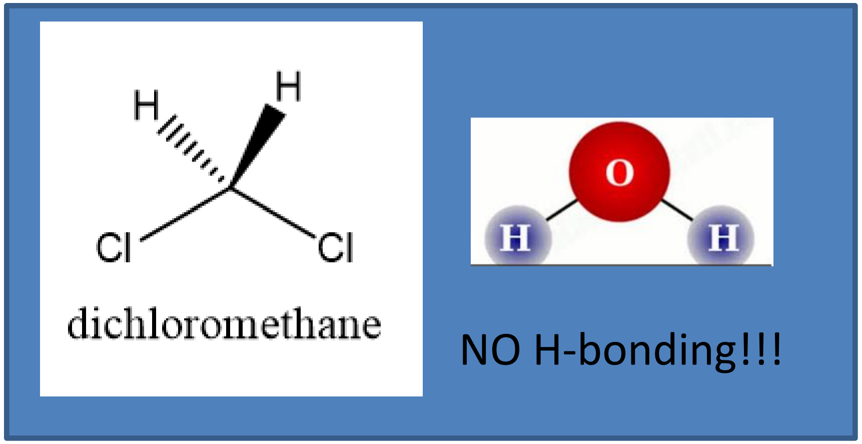

DCM and water

The "next" obvious question:

- If a molecule CAN H-bond with water…
  - will it be soluble in water?

Remember:
The overarching goal of ALL 3 experiments is to find relationships between molecular structure and water solubility.

Part B

- Goal - To develop general guidelines or 'rule of thumb' to *further* help predict water solubility for molecules that contain H-bonding groups.
- BQ: If a molecule CAN H-bond with water, will it be soluble in water?

- methanol
- ethanol
- 1-propanol
- 1-butanol
- 1-pentanol
- 1-hexanol
- 1-octanol

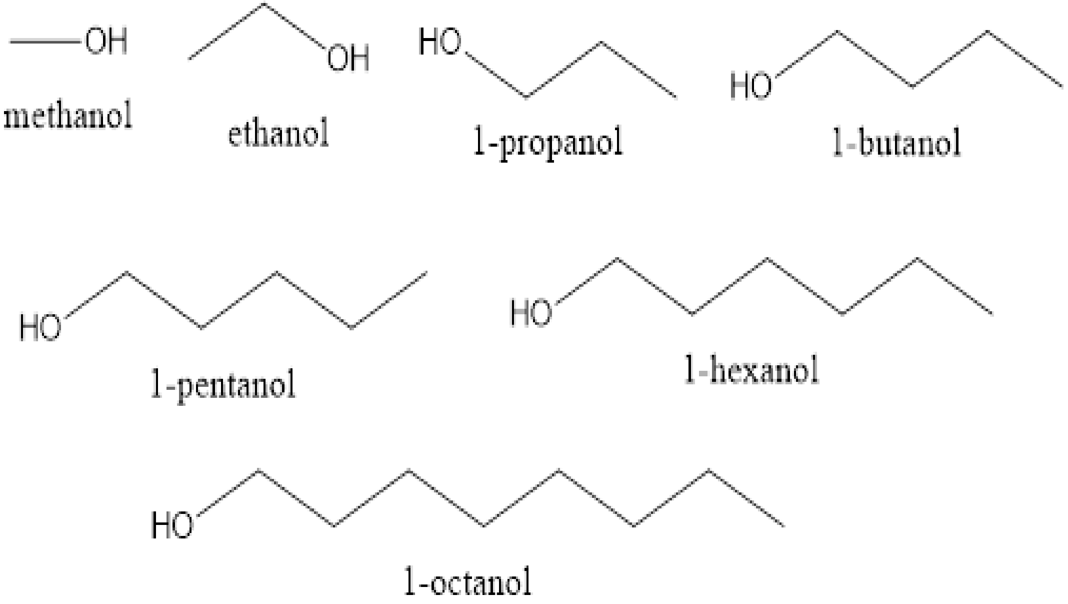

Part B: Procedural Information

1. Seven small vials were prepared for you
2. 1 mL of water was added to each
3. 25 drops of each alcohol added to a vial
4. The resulting solution was mixed well

Experimental Overview

Investigate the solubility of a series of alcohols in water in order to develop a solubility "rule of thumb".

Identify variables:

•**Independent variable** (what varies?)

•**Dependent variable** (what is measured?)

•**Controlled variable(s)**

6 replicates

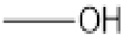

methanol

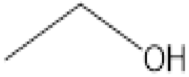

ethanol

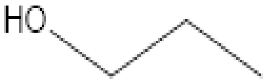

1-propanol

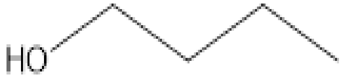

1-butanol

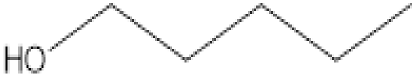

1-pentanol

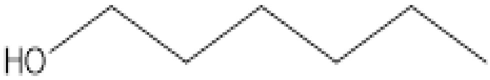

1-hexanol

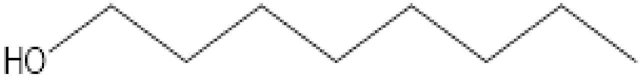

1-octanol

Experimental Overview

Investigate the solubility of a series of alcohols in water in order to develop a solubility "rule of thumb".

Identify variables:

- **Independent variable**
  - size of the carbon chain in an alcohol molecule
- **Dependent variable**
  - Two layers/miscibility (practical solubility) in water
- **Controlled variable(s)** –OH functional group and H-bonding with water, same amount of alcohol, same amount of water, temp, etc.

class provides replicates

Table 2. Solubility of 25 drops of a series of alcohols in water.

	solvent 1	solvent 2	Observations<br>no layers/2 layers, layer on top/bottom, ?	Inference<br>(solubility in water)<br>How soluble?
1	water	water		
2	water	methanol		
3	water	ethanol		
4	water	1-propanol		
5	water	1-butanol		
6	water	1-pentanol		
7	water	1-heptanol		
8	water	1-octanol		

***You have a set of the vials with these experiments in a small white box your hood.** You may pull them out and bring to the lab table to examine them.*

An ***observation*** is a descriptive statement about a natural phenomenon.

An ***inference*** is an interpretation of an observation.

RESULTS

**creating meaning from the data*

- water-miscible alcohols in the series

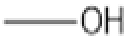
methanol

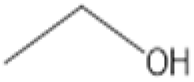
ethanol

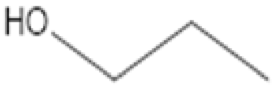
1-propanol

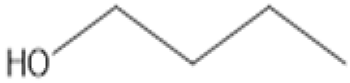
1-butanol

**predominant IMFs based on experimentation?*

Somewhat soluble/somewhat insoluble

- water-immiscible alcohols in the series

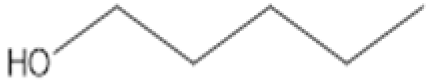
1-pentanol

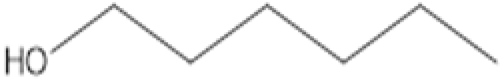
1-hexanol

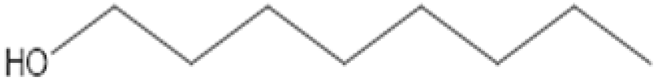
1-octanol

**predominant IMFs based on experimentation?*

Bring in some “nuance”

- ***High solubility in water***
- ***Low solubility in water***
- ***Mostly insoluble in water***
- ***Insoluble in water***

NOT simply:
Water soluble?
Water insoluble?

****NUANCED*

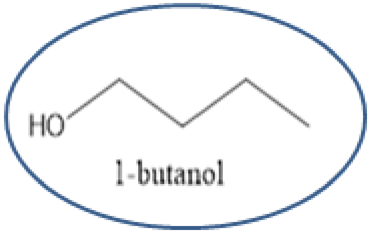

Predominant IMFs

- Identify all IMFs between molecules of interest (here solvent and solute).
- Decide which IMFs are PREDOMINANT
  - If applicable – sometimes there is no one predominant IMF
- **Sometimes the strongest IMFs are not the most predominant**

***Predominant = having superior influence, the most frequent or common, MAJORITY**

‘Rule of thumb’ to predict water solubility?

- *5-6 C rule of thumb*
  - *Even if they contain H-bonding functional groups…..**molecules with more than 5-6 C atoms are often insoluble in water***
  - **the more C atoms in a molecule, the more surface area, so…. LDFs predominate* (majority)

*record this finding in your handout

When predicting water solubility based on molecular structure:

A) If an organic molecule cannot H-bond with water, it cannot dissolve in water. *LIKE IMFs dissolve LIKE IMFs.*

B) Even if molecules contain H-bonding groups…consider the # of C atoms (molecular size/surface area) and the predominant (*majority*) IMFs.

**sometimes the ability to H-bond is outweighed, sometimes not*

Concept check: sucrose? (table sugar)

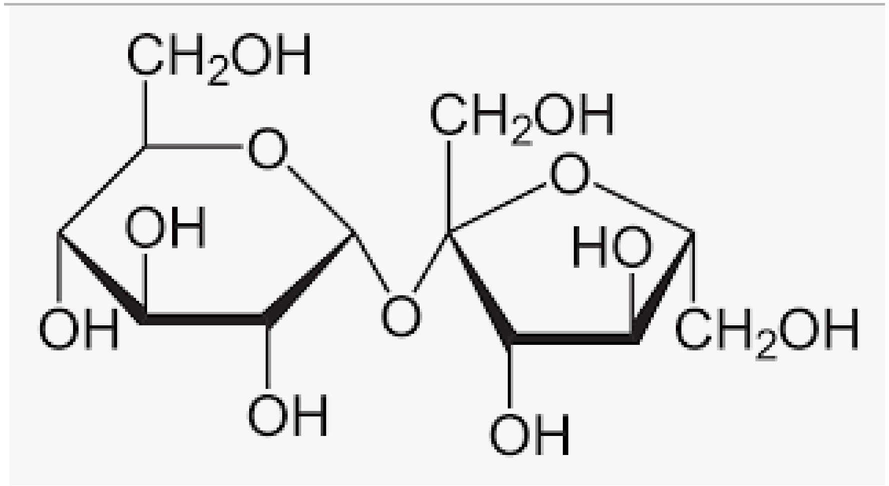

*RECORD as evidence in your table:
sucrose is highly water soluble

Picture in your mind…. sucrose dissolving in water

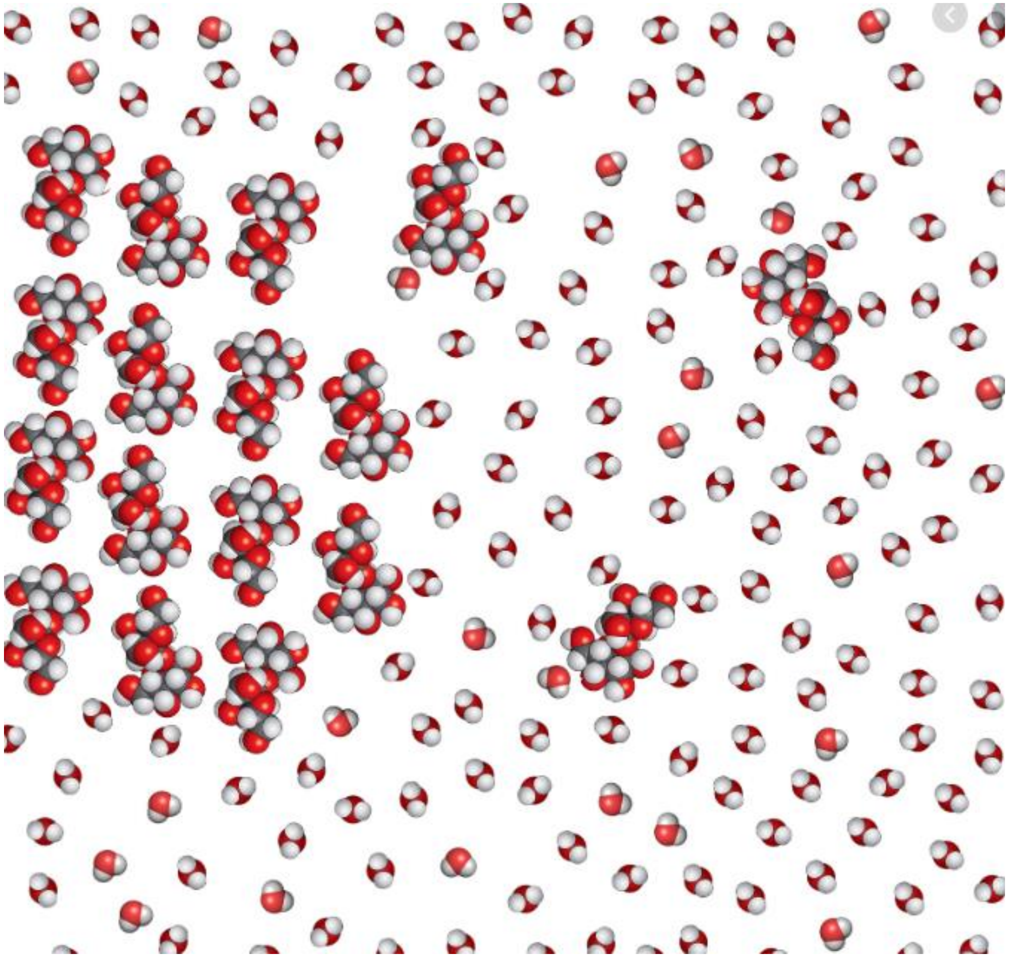

Physical process
IMFs only

NO COVALENT BONDS BROKEN

*This is the MOLECULAR level of the chemistry triangle

Dissolving is NOT a chemical change

Chemical vs. Physical Change

- **Physical Change**: A change that can occur without changing the identity of the substance.
  - Ex. Solid, Liquid, Gas (Phase change)
- **Chemical Change**: Process by which a substance becomes a new and different substance

No change in molecular structure

Change in molecular structure or atoms making up structure

*NO covalent bonds broken within sucrose

‘Rule of thumb’ to predict water solubility *revised?

- *5-6 C rule*
  - *Molecules with more than 5-6 C atoms are usually insoluble in water – unless there are a **nearly balanced** number of highly polar H-bonding sites.*

  - *WHY?*The more C atoms in a molecule, the larger the surface area, the more LDFs are the predominate IMF unless there are a nearly balanced number of highly polar H-bonding sites.*

*record revisions in your handout

Circling back to ethyl acetate:

Water insoluble	ethyl acetate **top*	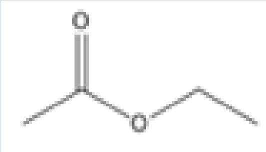	H-bonding<br>dip-dip<br>LDF

What's going on?

An even more nuanced approach

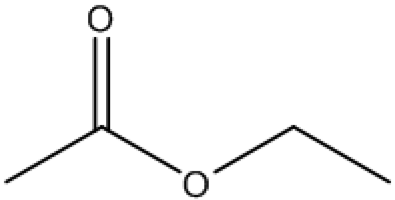

VS

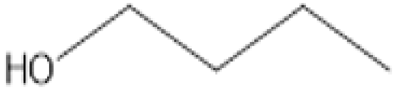

1-butanol

ethyl acetate

Not miscible with water (two layers formed)…

- *Although the amounts of each liquid added to the test tube were equal, one layer was slightly bigger….*

What does this indicate?

Miscible with water…but not completely (a tiny layer of butanol).

Less miscible than shorter chain alcohols and more miscible than longer carbon chain alcohols

An even more nuanced approach

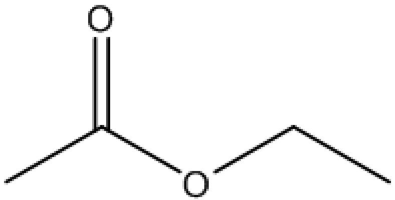

ethyl acetate

**mostly insoluble in water*

Why?

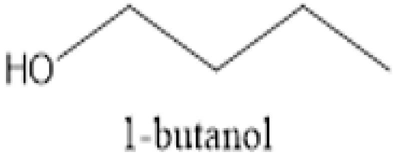

** Low solubility in water*

H-bond donors/H-bond acceptors

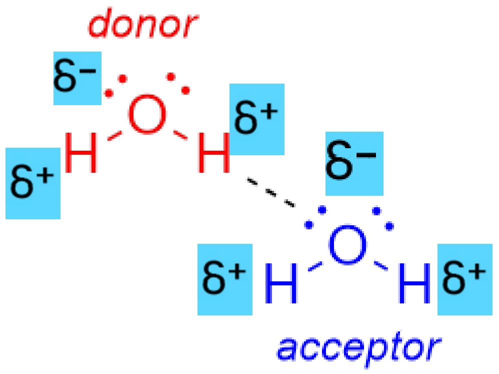

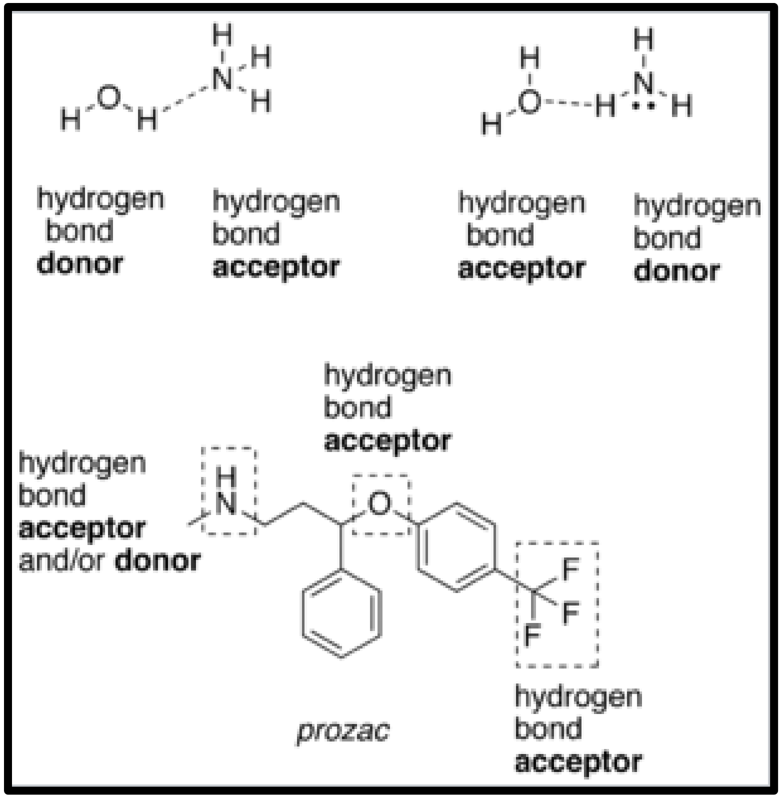

- donors are automatically acceptors
- acceptors can only accept

'Rule of thumb' – H-bond donors/acceptors

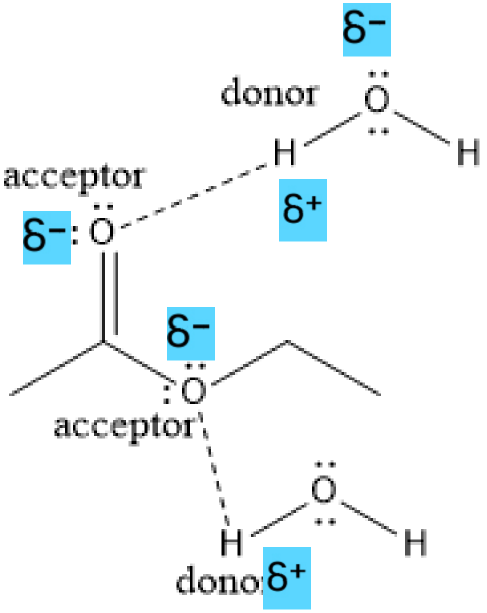

H-bond donating groups create **twice as many H-bonds** between the solute and water and **tend to increase water solubility.**

H-bond accepting groups **form fewer H-bonds**. **A molecule with only H-bond acceptors has fewer favorable H-bonding interactions** between the solute and water.

An even more nuanced approach

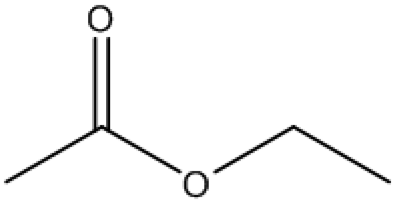

ethyl acetate

**mostly insoluble in water*

Why?

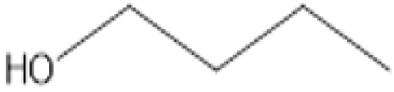

1-butanol

** Low solubility in water*

Concept check:

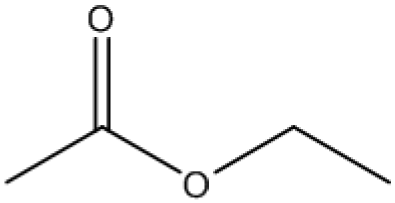

ethyl acetate

Why?

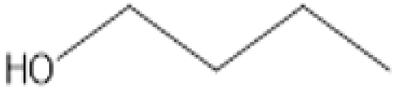

1-butanol

H-bond accepting groups

Forms fewer H-bonds with water, *predominant IMFs dip/dip & LDF*

**mostly insoluble in water*

H-bond donor & acceptor groups

Forms more H-bonds with water, *no predominant IMF (balanced)*

** low solubility in water*

Certain solubilities you observed today can help you remember the “rules of thumb”

- Sucrose

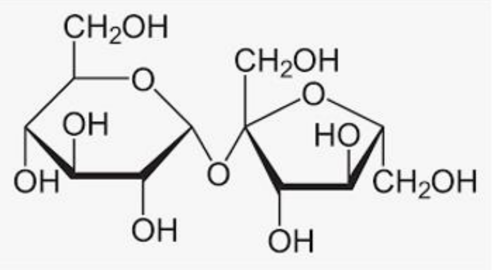

high solubility

- butanol

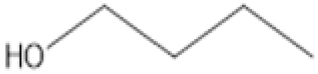

1-butanol

low solubility **what did you see*

- ethyl acetate

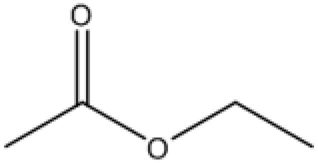

mostly insoluble*what did you see

- DCM (dichloromethane)

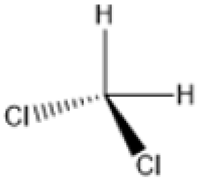

dichloromethane (DCM)

insoluble

*Make sure and record these…

you will make comparisons to these to help you explain solubility predictions!

Part C:

Goal - To develop another guideline or 'rule of thumb' for predicting the water solubility of charged ions and neutral molecules.

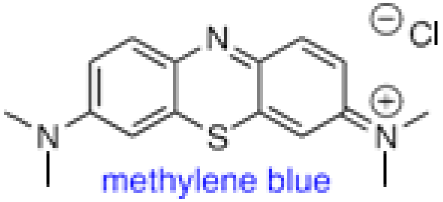

*polyatomic ion

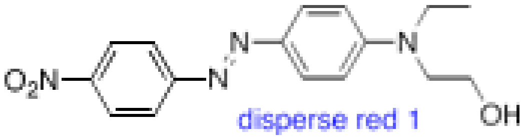

*neutral molecule

THINK/PAIR/SHARE with your team

FIRST THINGS FIRST….

Is methylene blue a cation or anion?

methylene blue

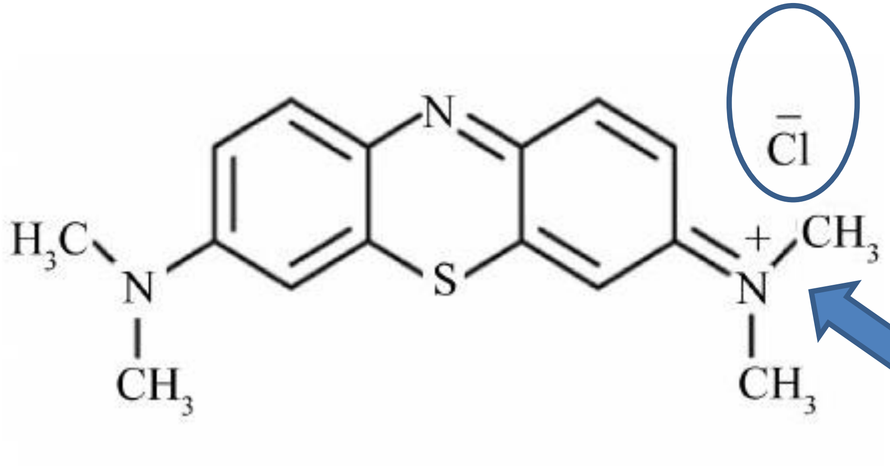

Just a counterion

Pay attention to the formal charge !
This is a polyatomic ion.

A **counterion** is the **ion** that accompanies an **ionic** species in order to maintain electric neutrality.

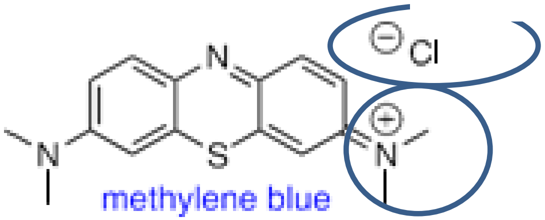

*polyatomic ion

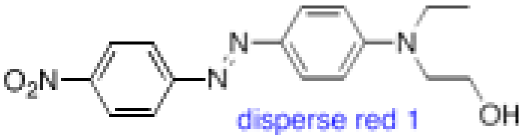

*neutral molecule

Experimental Overview: We will dissolve a small sample of dye in each solvent or solvent system. If a dye is soluble…we will see the color in the solvent. If a dye is insoluble…we will not see the color in the solvent.

What will you see if the red dye is soluble?

	**solvent 1**	**solvent 2**	**Observations** (color of solution, *if two solvents: identify top/bottom and color)	**Conclusions** solubility of the dyes
1	water	-none-		
2	ethanol	-none-		
3	ethyl acetate	-none-		
4	water	ethyl acetate		
5	water	DCM		

What will you see if the blue dye is soluble?

What will you see if both dyes are soluble?

Part C:

Goal - To develop a 'rule of thumb' for predicting the water solubility of charged (ionic) species and neutral molecules.

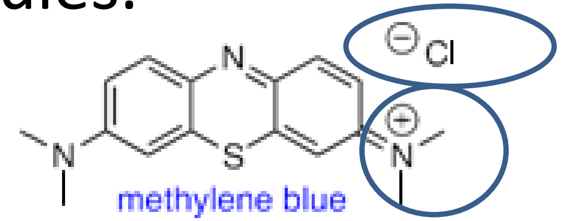

*polyatomic ion

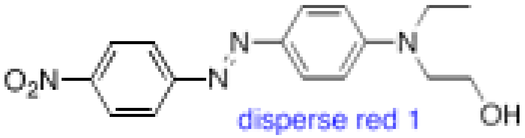

*neutral molecule

Identify variables:

•**Independent variable** (what varies)
structure/polarity/IMFs of solvents

•**Dependent variable** (what is measured)
dye solubility

•**Controlled variable(s)** amounts of solvents, amounts of dye mix, temp, etc.

VS

Identify variables:

•**Independent variable** (what varies)
structure/polarity/IMFs of solvents

•**Dependent variable** (what is measured)
dye solubility

•**Controlled variable(s)** amounts of solvents, amounts of dye mix, temp, etc.

Part C: 20 min

Table 3. Solubility of dyes in various solvents.

	solvent 1	solvent 2	Observations (color of solution, *if two solvents: identify top/bottom and color)	Inference- solubility of the dyes
1	water	-none-		
2	ethanol	-none-		
3	ethyl acetate	-none-		
4	water	ethyl acetate		
5	water	DCM		

What is the pattern or trend you OBSERVE?

What is difference in solvent polarity and dye solubility for the charged ionic dye vs the nonpolar molecular dye?

What is the pattern or trend you OBSERVE?

What is difference in solvent polarity and dye solubility: ionic dye vs the nonpolar molecular dye?

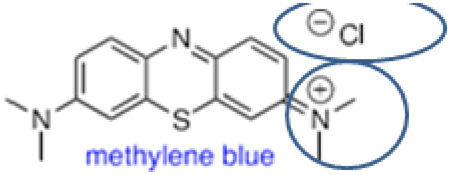

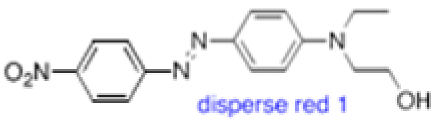

	MB charged cation	DR nonpolar molecule
soluble	Water<br>ethanol	ethyl acetate<br>DCM
insoluble	ethyl acetate<br>DCM	water
		**only a small amount soluble in ethanol*

RESULTS

**creating meaning from the data*

water-miscible dye

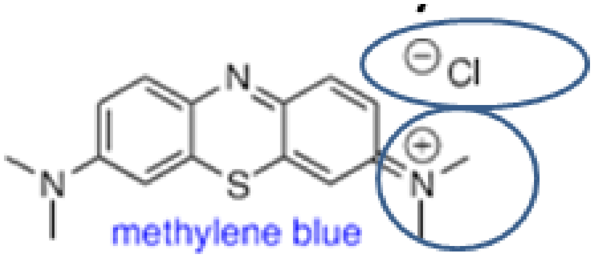

***polyatomic ion**

water-immiscible dye

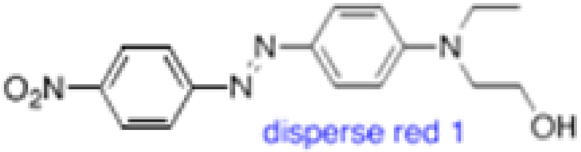

***neutral molecule**

The methylene blue (charged cation) is soluble in water and solvents that are soluble in water.

The methylene blue (charged cation) is INSOLUBE in solvents that are insoluble in water.

Solubility guideline from Part C:

Even if a structure has many C atoms, it may have greater water solubility due to ion –dipole forces.

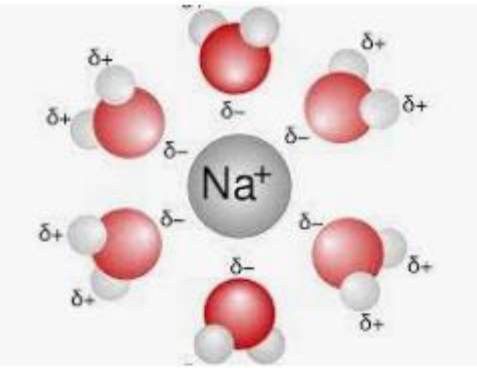

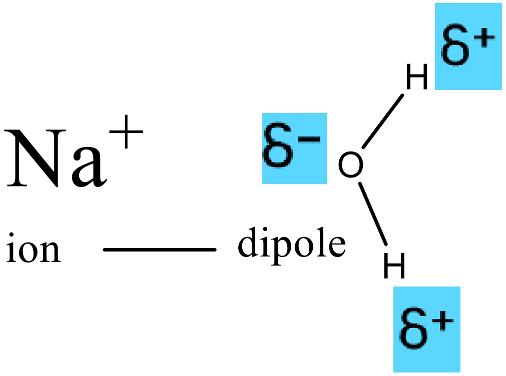

Ion–Dipole Attraction

- In a mixture, ions from an ionic compound are attracted to the dipole of polar molecules.
- The strength of the ion–dipole attraction is one of the main factors that determines the solubility of ionic compounds in water.

Ion-dipole forces

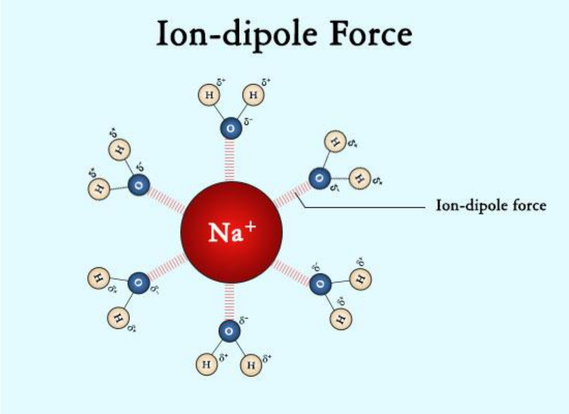

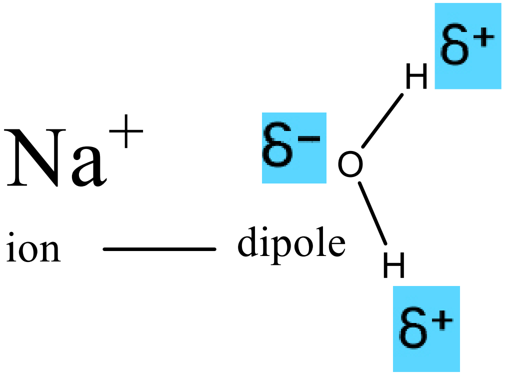

- An **ion–dipole** force consists of
  an **ion and a dipole from a polar molecule interacting**.
- **Ion-dipole** forces are **stronger** than **dipole-dipole** interactions because the charge of any **ion** is much greater than the charge of a **dipole** moment.
- **Ion-dipole** forces are **stronger** than hydrogen bonding, however, these intermolecular **ion-dipole** forces are much weaker than covalent or **ionic** bonds.

Picture in your mind… NaCl dissolving in water

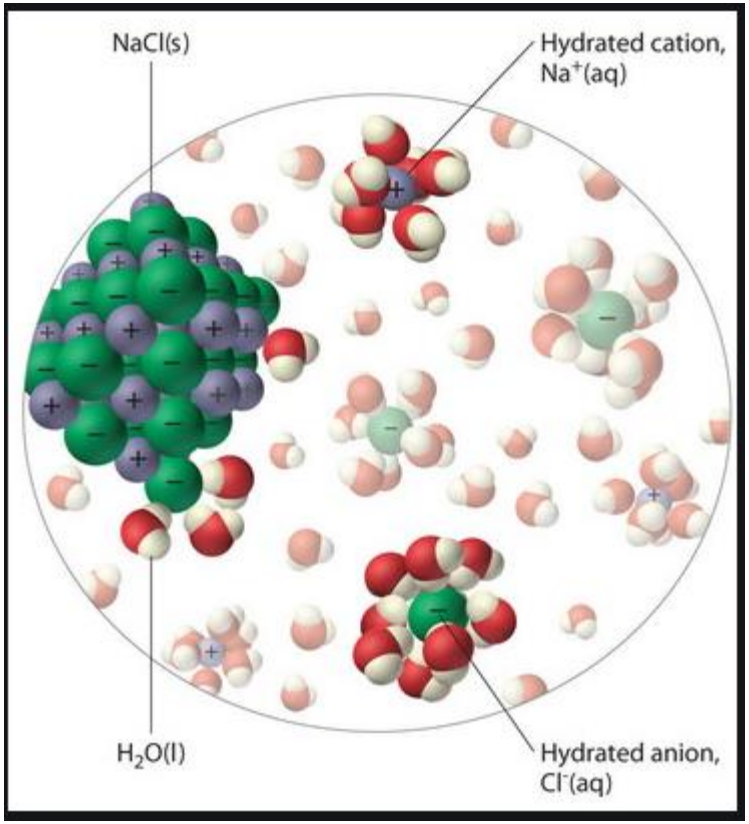

*this type of visualization is the MOLECULAR level of the chemistry triangle.

Chemistry Triangle

Observable/Symbolic/Molecular levels

The most important learning goal for the chemistry curriculum is to develop a molecular perspective.

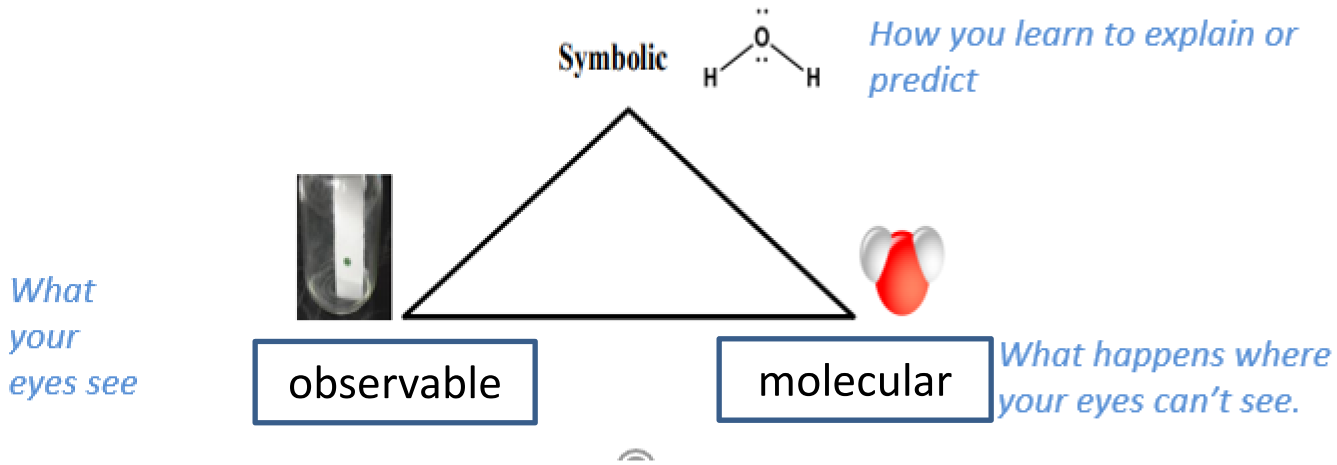

*Intentionally connecting all three levels is critical for your cognitive development as a chemistry student.

This is what we mean by developing "a molecular perspective"

Thinking on the Molecular level

- Many students struggle with understanding the “molecular level"
- It is how you think in your head..how you “see” the molecules behaving.
- This is the part of the triangle that novices mostly ignore. Practice! Create a VISION of the molecular level EVERY time you think about the observable.
- It's a framework for thinking about the molecular level that helps you understand how/why the macro scale behavior exists.

How is solubility like a high school cafeteria?

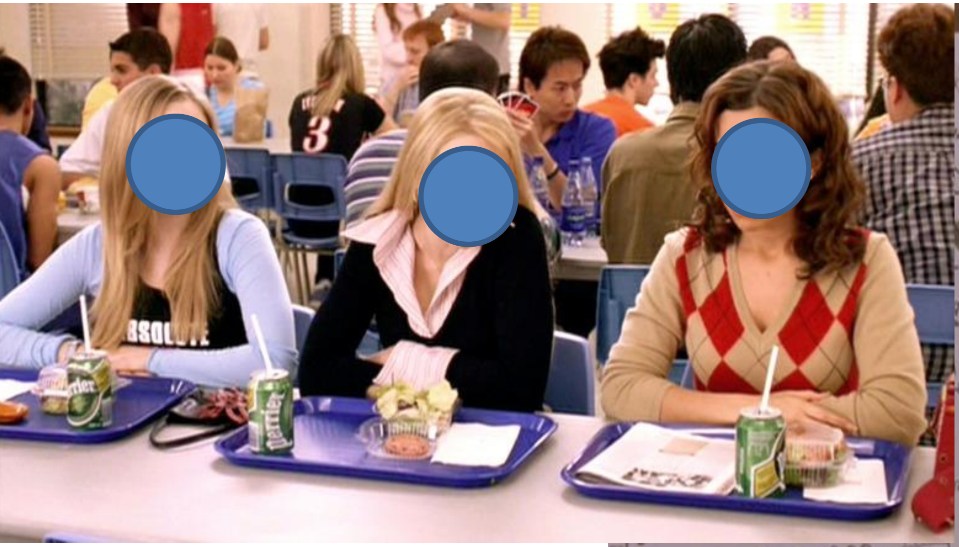

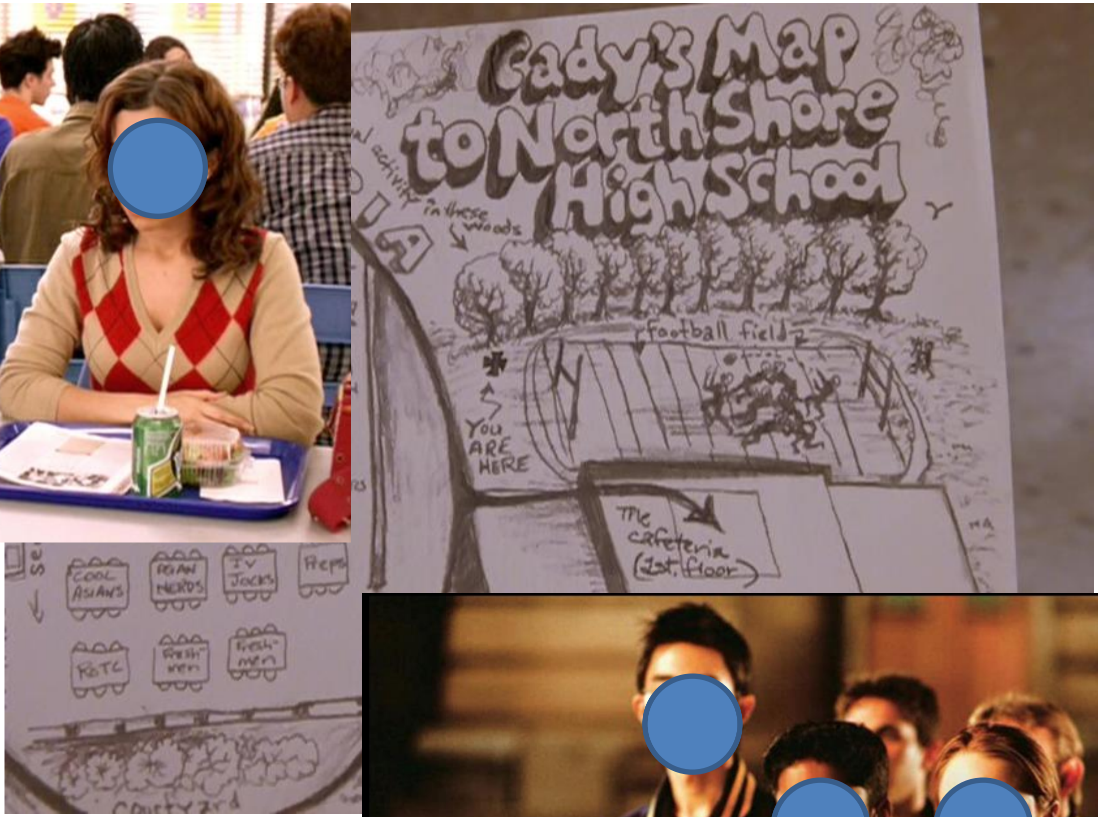

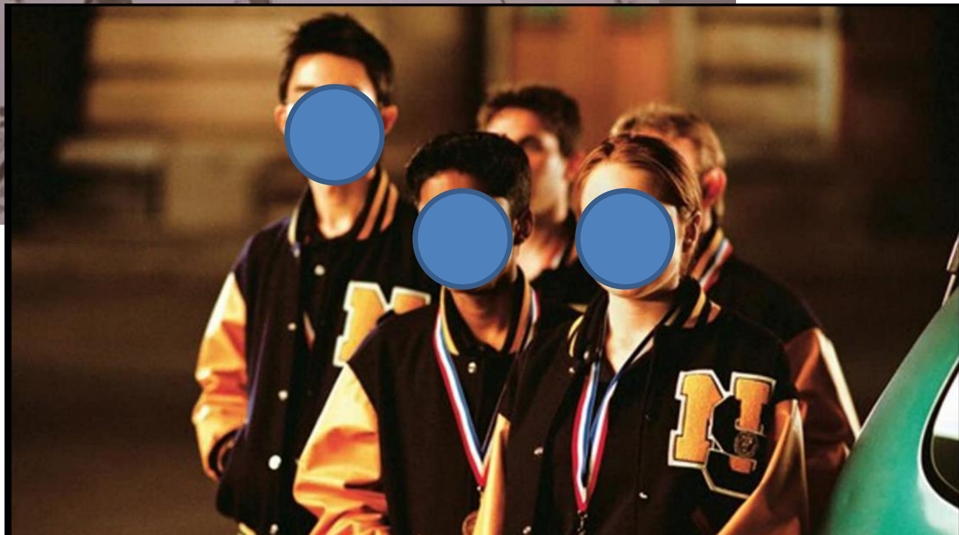

*record in your handout

What did you discover today?

When predicting water solubility based on molecular structure:

A) If an organic molecule cannot H-bond with water, it will not be miscible with water. ***like IMFs dissolve like IMFs***

B) 5-C Rule: Even if molecules have H-bonding groups, consider the # of C atoms (molecular size) and the predominant IMFs.**sometimes the ability to H-bond is outweighed, sometimes balanced … *H-bond acceptors weaker IMFs than H-bond donors, something to look at when there are 4-5 C*

C) Even if a structure is large (#C atoms), it can have greater water solubility if it is an ion, due to ion-dipole forces between the solute and water (the solvent). **Charged species (ions) have the strongest IMFs with water (due to ion-dipole forces)*

IMF concepts *are* not *going away*…

The important concepts learned today will be used again:

- ALL semester in 202L
- All semester in 203L
- All semester in 204 lecture and 204L
- Biology
- Biochemistry
- Human Anatomy/Physiology
- and beyond…

in increasingly sophisticated ways!

Beginning Question: Predict the water solubility of the chemotherapy drug :

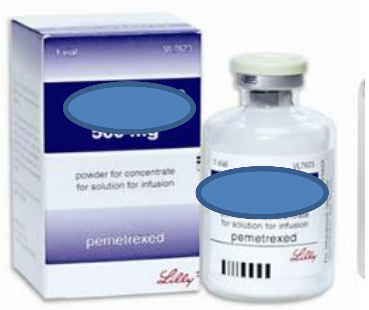

- ***High solubility in water***
- ***Low solubility in water***
- ***Relatively insoluble in water***
- ***Insoluble in water***

*This is a prediction. There is no one absolute “right” answer.

However, to show that you understand the concepts involved, your novice predictive claim must be only one away from an expert predictive claim.

**Your JUSTIFICATION is the most important part.

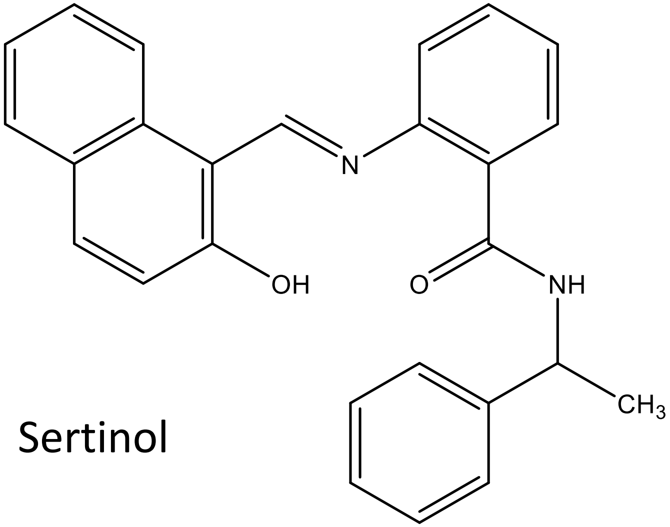

Sertinol

Remember the molecules we observed today that fit each category

- High solubility - sucrose

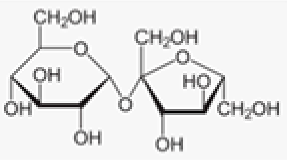

- Low solubility - butanol

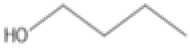

1-butanol

- Mostly insoluble- ethyl acetate

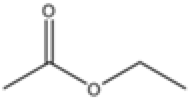

- Insoluble – DCM

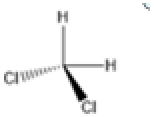

dichloromethane
(DCM)

Use EVIDENCE…compare the solubility to what you observed today in lab.

Is it water soluble?

- Finally, you must also think on the molecular level. Consider the totality of the shape of the drug and envision in your mind the way that water molecules might or might not be able to surround the drug to allow it to dissolve.
- Ask yourself –
  - ***Can water fully surround the molecule?***
  - ***Will the IMFs <u>between</u> water and the drug outweigh the favorable IMFs between-***
    - ***the water molecules?***
    - ***the drug molecules?***
