# Supplementary material for: Cultivating a Meaningful Application of IMFs through Backward Laboratory Course Design
Source: J Chem Educ. 2024 May 8;101(6):2331–42. doi: 10.1021/acs.jchemed.3c00810 (PMC11171254; doi:10.1021/acs.jchemed.3c00810)
Supplement: Supplementary file 9 — ed3c00810_si_009.pdf [file ed3c00810_si_009.pdf]

# Cultivating a Meaningful Application of IMFs Through Backward Laboratory Course Design

Brenda B. Harmon[a]*, Deepika Das[a], Annette W. Neuman[a], Simbarashe Nkomo[a], Nichole L. Powell[a], Austin Scharf[a]

[a] Department of Chemistry, Oxford College of Emory University, Oxford, GA 30054, United States
*Email: bharmon@emory.edu

## Solubility Lab Instructor Notes & Timeline

**~5 min** powerpoint intro to the lab session (through Taxol)

**~10 min** set up **Part A** -THINK – PAIR-SHARE have students individually rank solvents in polarity and make solubility predictions in their notebooks, then discuss in pairs, then within their 4 person team. Discuss what theoretical frameworks/mental models they used **Summarize these on the board (cross off unhelpful ones as the lab session evolves *especially "polarity"explain that it is not enough).*

**~5 min** students work in groups to identify variables - lead class discussion, reveal variables

**~20 min** Perform Part A work in groups of 4.

**~5min** Pool class data - a table on the overhead (first group to finish tells you the obs/inference – make sure all groups agree on the data *usually some mess up on identifying which layer is top/bottom -*this is good problem solving for them*) Students repeat questionable data

**~5-min** Discussion about usefulness of different frameworks for predicting solubility.

For which predictions do polarity and like dissolve like fall short? **Can IMF help to predict**?

FINISH PART A by 1 hr mark *(it usually takes ~45 minutes)*

---

**Part B** - the students do not perform, but they must record observations in a table. Set it up using the ppt slides, identify variables.

**~10- min** set up

**~ 5-10 mins** students look at the samples.

**~5-10 min** Pool class data in the provided table. Have them summarize or create meaning from the observations.

~15 minutes The concept checks/ H-bond acceptor/donor /pulling it all together

FINISH PART B by the 1 hr 50 min mark

---

## Part C

**~5 min** Students think/share whether MB is a cation or anion

**~ 5min** Explain the experiment to them. Explain the variables.

**20 min** Have them perform the experiment. *Make sure they use a tiny amount on the end of the plastic stick, dip it into the little vial, re-use for all trials. Pool class data in table/repeat questionable data

**~5 min** *Have them describe the **observations** and try to identify a pattern in observations **before** they try to explain where their eyes can't see. (if necessary, lead them to MB soluble in water and water soluble solvents, disperse red soluble in water insoluble solvents).

**~5-10 min** pull it ALL together

***~20 min*** *Have them work together to answer the beginning question.* Their explanation/justification using the concepts from the lab is most important part. Walk around and see where they are and give feedback/answer questions. Lead them to use the KEY molecules as a general guide.

*THE EXACT RIGHT ANSWER DOES NOT EXIST***As long as their (novice) prediction is no more than one away from your (expert) prediction they are okay as long as they can JUSTIFY/EXPLAIN their answer using correct concepts/vocab/molecular level drawings.*

# Student Workers will CLEAN UP. Your students will likely NOT have time.

In order to helps students with their predictions/justifications:

- ***High solubility in water***
- ***Low solubility in water***
- ***Mostly insoluble in water***
- ***Insoluble in water***

Get them to give context for their choice...more soluble than ethyl acetate? (why), less soluble than sucrose? (why)

Whichever they choose...ask them to explain why they did not choose the two options on either side???

You must show students how to think through this on all three levels of the triangle.
